# Exploring C-to-G and A-to-Y Base Editing in Rice by Using New Vector Tools

**DOI:** 10.3390/ijms23147990

**Published:** 2022-07-20

**Authors:** Dongchang Zeng, Zhiye Zheng, Yuxin Liu, Taoli Liu, Tie Li, Jianhong Liu, Qiyu Luo, Yang Xue, Shengting Li, Nan Chai, Suize Yu, Xianrong Xie, Yao-Guang Liu, Qinlong Zhu

**Affiliations:** 1 State Key Laboratory for Conservation and Utilization of Subtropical Agro-Bioresources, College of Life Sciences, South China Agricultural University, Guangzhou 510642, China; dong@scau.edu.cn (D.Z.); xinaxin@stu.scau.edu.cn (Z.Z.); liuyuxin@stu.scau.edu.cn (Y.L.); liutaoli@stu.scau.edu.cn (T.L.); litie@stu.scau.edu.cn (T.L.); liujianhong@stu.scau.edu.cn (J.L.); qiyuluo@scau.edu.cn (Q.L.); xueyang@stu.scau.edu.cn (Y.X.); lishengting@stu.scau.edu.cn (S.L.); chinan@scau.edu.cn (N.C.); ysz@stu.scau.edu.cn (S.Y.); xiexianrong@scau.edu.cn (X.X.); ygliu@scau.edu.cn (Y.-G.L.); 2 Guangdong Laboratory for Lingnan Modern Agriculture, Guangzhou 510642, China

**Keywords:** CGBE, ABE8e-EndoV, CBE, ABE, CRISPR/Cas9, rice

## Abstract

CRISPR/Cas9-based cytosine base editors (CBEs) and adenine base editors (ABEs) can efficiently mediate C-to-T/G-to-A and A-to-G/T-to-C substitutions, respectively; however, achieving base transversions (C-to-G/C-to-A and A-to-T/A-to-C) is challenging and has been rarely studied in plants. Here, we constructed new plant C-to-G base editors (CGBEs) and new A-to-Y (T/C) base editors and explored their base editing characteristics in rice. First, we fused the highly active cytidine deaminase evoFENRY and the PAM-relaxed Cas9-nickase variant Cas9n-NG with rice and human uracil DNA N-glycosylase (rUNG and hUNG), respectively, to construct CGBE-rUNG and CGBE-hUNG vector tools. The analysis of five NG-PAM target sites showed that these CGBEs achieved C-to-G conversions with monoallelic editing efficiencies of up to 27.3% in T_0_ rice, with major byproducts being insertion/deletion mutations. Moreover, for the A-to-Y (C or T) editing test, we fused the highly active adenosine deaminase TadA8e and the Cas9-nickase variant SpGn (with NG-PAM) with *Escherichia coli* endonuclease V (EndoV) and human alkyladenine DNA glycosylase (hAAG), respectively, to generate ABE8e-EndoV and ABE8e-hAAG vectors. An assessment of five NG-PAM target sites showed that these two vectors could efficiently produce A-to-G substitutions in a narrow editing window; however, no A-to-Y editing was detected. Interestingly, the ABE8e-EndoV also generated precise small fragment deletions in the editing window from the 5′-deaminated A base to the SpGn cleavage site, suggesting its potential value in producing predictable small-fragment deletion mutations. Overall, we objectively evaluated the editing performance of CGBEs in rice, explored the possibility of A-to-Y editing, and developed a new ABE8e-EndoV tool, thus providing a valuable reference for improving and enriching base editing tools in plants.

## 1. Introduction

Cytosine base editors (CBEs) and adenine base editors (ABEs) based on CRISPR/Cas9 variants have applications in animal and plant functional genomics and genetic engineering. From BE1 to evoBE4max of the different generations of CBEs [1], researchers have mainly paid attention to improving C-to-T substitution efficiency and developing a series of effective CBEs, such as PhieCBEs [2], hA3A-PBE [3], Anc689BE4max-nCas9 [4], and DisSUGs [5]. From TadA7.10 to TadA8e [6], researchers have improved A-to-G substitution efficiency and developed efficient ABEs, such as PhieABEs [7], rBE53~rBE65 [8], SpG-ABE8e and SpRY-ABE8e [9,10,11], PTG-ABE8e [12], and ABEmax-nCas9 [4]. Efforts have been made to improve the efficiency of ABEs and CBEs for achieving high-efficiency and stable base editing in plant genomes, and these tools have been widely used in gene function research and crop genetic improvement [13,14,15,16,17,18].

The currently available base editors can implement C-to-T/G-to-A and A-to-G/T-to-C substitutions; however, achieving base transversion remains challenging. For CBEs, cytosine (C) is deaminated to uracil (U), and U is recognized as thymine (T) at the DNA level, leading to C-to-T substitution after DNA repair and replication [19]. However, via the base excision repair (BER) pathway in eukaryotic cells, the U bases of U:G mismatches can be easily removed by uracil DNA N-glycosylase (UNG), thus resulting in apurinic/apyrimidinic (AP) sites. The AP sites are hydrolyzed by AP lyase via DNA repair to achieve C-to-G/C-to-A transversions and insertions/deletions (indels). Based on the principle of BER, the uracil DNA glycosylase inhibitor (UGI) domain in the CBE system is replaced by the UNG domain, and high-efficiency C-to-G transversion is produced in mammalian cells [20,21]. Although studies have evaluated CGBE editing in protoplasts and stable transformations in rice and poplar [22,23], the target sites and data are insufficient to objectively assess the editing performance of CGBE in plants.

For ABEs, adenine (A) in the editing window is deaminated to inosine (I), and I is recognized as guanine (G) at the DNA level, ultimately achieving A-to-G substitution after DNA repair and replication [19]. Because of the lack or low activity of glycosylase on the I base in eukaryotic cells, the ABE system contains few A-to-Y (C or T) or indel byproducts [6,7]. However, a study indicated that cells are subjected to natural hydrolysis and nitrosative stress upon exposure to endogenous and/or exogenous agents, leading to the deamination of A to I and I:T mismatches that stimulate both BER and alternative excision repair (AER) pathways [24]. In the BER pathway, alkyladenine DNA glycosylase (AAG) catalyzes the cleavage of the glycosidic bond of inosine to remove the I base from the DNA strand [24,25]. In the AER pathway, endonuclease V (EndoV) hydrolyzes the second phosphodiester bond located at 3′ to inosine in the DNA or RNA strand [24,26,27,28]. In the *E. coli* model, an EndoV-dependent AER pathway for the removal of deoxyinosine from DNA has been identified. However, the AER pathway has not yet been identified in mammalian cells. According to a biochemical investigation of mammalian EndoV, it possesses considerable inosine 3′ endonuclease activity on inosine-containing single-stranded DNA (ssDNA) and poor activity on double-stranded DNA (dsDNA) [24,26,27,28]. Studies on the BER and AER pathways based on I:T mismatches in the ABE system have been limited. Therefore, we integrated hAAG and EndoV by using ABE tools and explored whether they could achieve A-to-Y or A-to-indel editing.

In this study, based on the U:G mismatch BER pathway, we used rice UNG (rUNG) and human UNG (hUNG) to construct PevoFRNY-Cas9n-NG-rUNG (CGBE-rUNG) and PevoFRNY-Cas9n-NG-hUNG (CGBE-hUNG), respectively, and evaluated their C-to-G editing performance in rice. In addition, based on the I:T mismatch repair pathway, we used human AAG (hAAG) of the BER pathway and *E. coli* EndoV (EndoV) of the AER pathway to construct ABE8e-hAAG and ABE8e-EndoV vectors and explored their editing performance of A-to-Y transversions or indels in rice.

## 2. Results

### 2.1. CGBE-rUNG and CGBE-hUNG Achieved C-to-G Base Editing

**Figure 1:**
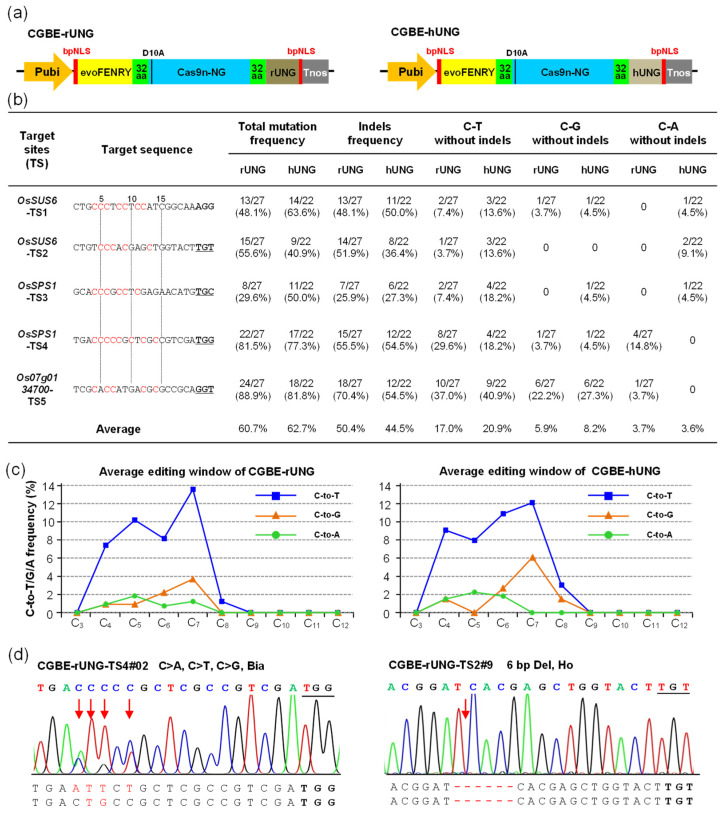
The editing efficiency and mutation types of CGBE-rUNG and CGBE-hUNG in T_0_ rice. (**a**) Structures of the CGBE-rUNG and CGBE-hUNG expression cassettes. evoFERNY, a cytosine deaminase with efficient C-T substitution; Cas9n-NG, an SpCas9 variant that recognizes NG-PAM and only has cleavage activity on the target strand; rUNG, uracil N-glycosylase from rice; hUNG, uracil N-glycosylase from humans. (**b**) The efficiency and mutation type of CGBE-rUNG and CGBE-hUNG in T_0_ rice, total mutations, and indel frequency were calculated by edited plants/total transgenic positive plants; for C-to-T, C-to-G, and C-to-A substitutions, base substitutions with indel alleles were eliminated. (**c**) The average editing window and editing efficiency of C-to-T/G/A substitution; the 20th base at the 5′ end of PAM is numbered C_1_. (**d**) Sanger sequencing chromatogram for some typical mutation types. Bia, biallelic mutation.

The amino acid sequences of UNGs are highly conserved between prokaryotes and eukaryotes; hence, UNGs from humans (1AKZ_A), rice (XP_015634353.1), Arabidopsis (NP_188493.1), and *E. coli* (EFN1850840.1) were screened by NCBI BLAST. We selected hUNG reported in mammalian cells and rUNG in plant cells as the candidates. We first optimized the nucleic acid sequences of hUNG and rUNG according to the plant codon preferences of rice, followed by chemical synthesis of these sequences (Sequence S1). PevoFRNY-Cas9n-NG, with high efficiency and broader genome-targeting protospacer-adjacent motifs (PAMs) (recognizes NG-PAM), was selected as the basic vector for further modification [2]. The 2xUGI domains of PevoFRNY-Cas9n-NG were replaced by hUNG and rUNG, respectively, to construct PevoFRNY-Cas9n-NG-rUNG (CGBE-rUNG) and PevoFRNY-Cas9n-NG-hUNG (CGBE-hUNG) (Figure 1a). CGBE-rUNG and CGBE-hUNG have a structure similar to that of the reported CGBE system [20]. Five NG-PAM targets from the rice genome with sgRNA were assembled into CGBE-rUNG and CGBE-hUNG to explore and compare their editing performance in rice. The overall editing efficiencies of CGBE-rUNG (29.6–88.9%) and CGBE-hUNG (40.9–81.8%) were measured in five targets, with the average editing efficiency being 60.7% and 62.7%, respectively (Figure 1b). However, the mutation types were mainly C-to-indels with both CGBE-rUNG (50.4%) and CGBE-hUNG (44.5%) editing (Figure 1b, Tables S1 and S2). We then calculated the C-to-T/G/A editing efficiency without indels, as indels generally cause nonsense mutations. To ensure the inheritance of the base-edited mutants, the alleles containing C-to-T/G/A substitutions with indels were excluded, such as CGBE-rUNG-TS1#06 and CGBE-rUNG-TS5#07 Allele 1 (A1) (Tables S1 and S2). The base editing results showed that the average C-to-T/G/A base editing efficiency of CGBE-hUNG (20.9%/8.2%/3.6%) was slightly higher than that of CGBE-rUNG (17.0%/5.9%/3.7%); however, both CGBE-hUNG and CGBE-rUNG base editing types were mainly C-to-T substitutions, and the C-to-G transversion average efficiency was less than 10% (Figure 1b). These results are inconsistent with those obtained in mammalian cells, in which CGBE systems significantly reduced C-to-T substitution and improved C-to-G transversion without excessive indel byproducts [20]. However, our results are almost consistent with those of studies on rice and poplar [22,23]. The allelic variations in CGBE-rUNG and CGBE-hUNG are mainly biallelic mutations and heterozygous mutations, with homozygous mutations in a small part (Figure 1d, Figures S1 and S2). The C-to-T editing window of CGBE-rUNG and CGBE-hUNG was mainly C_4_-C_7_, which is narrower than that of PevoFRNY-Cas9n-NG (C_3_-C_8_, extended to C_3_-C_10_) [2]. The C-to-G editing window of for CGBE-rUNG (C_4_-C_7_) was slightly narrower than that of CGBE-hUNG (C_4_-C_8_); however, the C-to-A window of CGBE-rUNG (C_4_-C_7_) was slightly wider than that of CGBE-hUNG (C_4_-C_6_) (Figure 1c and Figure S1).

To determine whether replacing the UGI domain with the UNG domain would increase the off-target effects, we selected off-target sequences from five targets of CGBE with a mismatch of ≤3 bases as candidate off-target sites for further sequencing analysis (Figure S3a) [29,30,31]. The sequencing results showed that among the 15 candidate off-target sites of CGBE, except for TS4-off2 of CGBE-hUNG (4.5%) with a discovered mutation, no mutations were found in other candidate off-target sites (Figure S3a). The results suggested that the candidate off-target sequence, which had more than two bases’ difference from the on-target sequence of the UNG-based CGBE system, also did not increase the off-target effects. Thus, in terms of the C-to-G editing window, editing efficiency, and product purity, the performance of hUNG was slightly superior to that of rUNG. Although the C-to-G efficiency of CGBE-TS5 was as high as 22.2% (rUNG) and 27.3% (hUNG), achieving C-to-G transversion without byproducts at most of the targets is a great challenge (Figure 1b).

### 2.2. A-to-Y Transversions Were Not Detected by ABE8e-EndoV and ABE8e-hAAG

**Figure 2:**
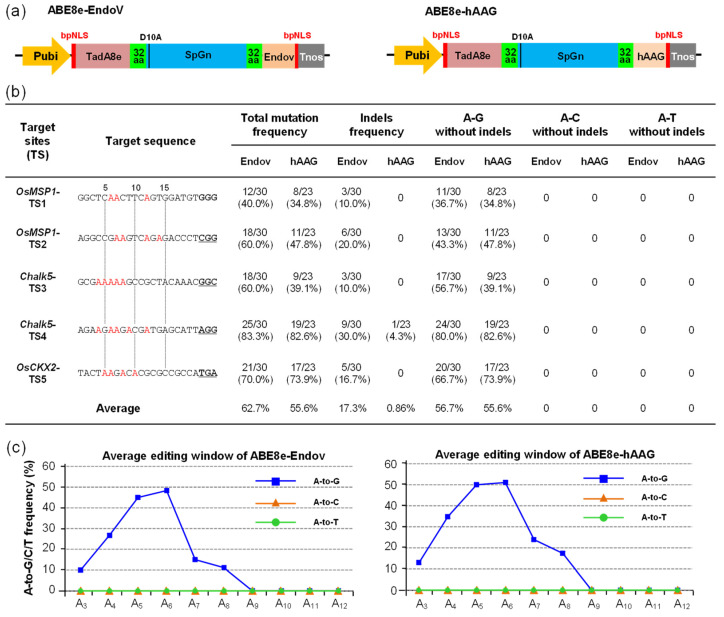
The editing efficiency and mutation type of ABE8e-EndoV and ABE8e-hAAG in T_0_ rice. (**a**) Expression cassette structures of ABE8e-EndoV and ABE8e-hAAG. TadA8e, adenine deaminase with efficient A-to-G substitution; SpGn, an SpCas9 variant that recognizes NG-PAM and inactivates the cleavage activity of non-target strands; EndoV, *E. coli* endonuclease V, which works in the inosine alternative excision repair (AER) pathway, hydrolyzes the second phosphodiester bond located at 3′ to inosine in the DNA strand; hAAG, human alkyladenine DNA glycosylase, which works in the inosine BER pathway, removes inosine, and generates an apurinic/apyrimidinic (AP) site. (**b**) The efficiency and mutation type of ABE8e-EndoV and ABE8e-hAAG in T_0_ rice, total mutations, and indel frequency were calculated by editing plant/total transgenic positive plants; for the A-to-G, A-to-C, and A-to-C substitutions, the base substitutions with indel alleles were eliminated. (**c**) Average editing windows and average editing efficiency of A-to-G/C/T substitution.

hAAG and EndoV play a role in the I:T mismatch BER and AER pathways; hence, we selected human AAG (hAAG) and *E. coli* EndoV (EndoV) to optimize codons and chemically synthesize their DNA sequences according to the codon preferences of rice (Sequence S2). The ABE8e-SpG developed by our team with high efficiency and broad targets (to recognize NG-PAM) was selected as the basic vector for further modification [7]. hAAG and EndoV were fused to the C-terminus of ABE8e-SpG to construct ABE8e-SpGn-EndoV (ABE8e-EndoV) and ABE8e-SpGn-hAAG (ABE8e-hAAG), respectively (Figure 2a). Five targets from the rice genome were selected to explore their editing performance; ABE8e-EndoV and ABE8e-hAAG exhibited stable editing efficiency in the five targets, and the overall average editing efficiency of ABE8e-EndoV (62.7%) was slightly higher than that of ABE8e-hAAG (55.6%). Moreover, the average A-to-G editing efficiency of ABE8e-EndoV (56.7%) and ABE8e-hAAG (55.6%) without indels was the same as that of ABE8e-SpG (59.7%), indicating that the addition of the hAAG and EndoV domains did not affect the deaminase activity of ABE8e-SpG [7]. In addition to A-to-G substitution, ABE8e-EndoV generated monoallelic indel mutations with an average efficiency of 17.3%, indicating that EndoV works on ABE system-mediated inosine repair. However, for ABE8e-hAAG, except for one indel mutation (4.3%) at TS4, the other mutants were A-to-G substitutions, which might be because of the low activity of hAAG. A-to-Y transversions were not detected in ABE8e-EndoV and ABE8e-hAAG, probably because I bases are strictly recognized as G at the DNA level or the activity of hAAG and EndoV is not sufficiently high (Figure 2b). The allelic variations in ABE8e-EndoV and ABE8e-hAAG were mainly biallelic and heterozygous mutations, with homozygous mutations in a small part (Figure S3b and Tables S2 and S3). The A-to-G editing window of ABE8e-EndoV and ABE8e-hAAG was concentrated in A_4_–A_7_ (extended to A_4_–A_8_). Moreover, ABE8e-EndoV and ABE8e-hAAG had narrower editing windows than hyABE8e-SpGn (A_4_–A_8_, extended to A_4_–A_11_) [7] (Figure 2c).

To evaluate whether fusing EndoV and hAAG domains would increase the off-target efficiency, we used the same off-target analysis method as that used for CGBEs and screened 13 candidate off-target sites (Figure S3b). The sequencing results showed that except for one mutant (TS2-off1: 3.3%) of ABE8e-EndoV with off-target mutation, no mutations were found in other candidate off-target sites (Figure S3b). The off-target analysis of ABE8e-EndoV and ABE8e-hAAG also indicated that the fusion of EndoV and hAAG with ABE8e did not result in a high frequency of off-target mutations.

### 2.3. ABE8e-EndoV Produces Predictable Small DNA Fragment Deletions

**Figure 3:**
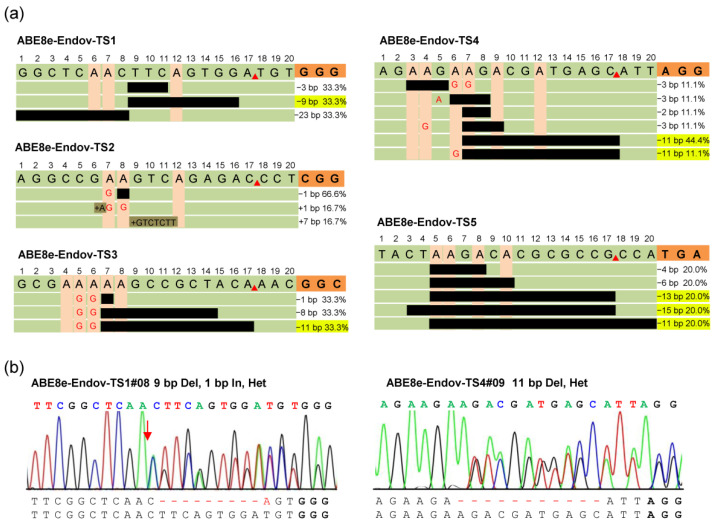
ABE8e-EndoV exhibits predictable small DNA fragment deletions. (**a**) ABE8e-Endov generates precise deletions from the 5′-deaminated A base to the SpGn nicking site. The proportion of deletions versus total indels is shown below PAM, and the uniform precise deletion ratio is highlighted in yellow; PAMs are highlighted in orange, and black bars represent the deleted nucleotide fragment. (**b**) Sanger sequencing chromatogram for some typical mutation types of ABE8e-Endov. Het, heterozygous mutation; Del, deletion; In, insertion.

CRISPR/Cas9 has HNH and RuvC nuclease domains, which cleave the target and non-target strands, respectively, leading to DNA double-strand breaks and random insertion and deletion mutations. In animal and plant genomes, several *cis*-acting elements composed of small-nucleotide fragments play a key role in the spatiotemporal-specific expression of genes [16]. CRISPR/Cas9 requires NGG-PAM recognition and random indel production, which is a challenge for functional studies of specific small nucleotide fragments. In this study, ABE8e-EndoV was detected with an average of 17.3% indel mutations. Further analysis of these indels indicated that ABE8e-EndoV generated the precise deletion of small fragments from 5′-deaminated A bases to the SpGn nickase cleavage site in TS1 (33.3%), TS3 (33.3%), TS4 (55.5%), and TS5 (60%) (Figure 3a). The deletion window mainly focuses on the A_5_-A_8_ to the SpGn cleavage site, leading to the deletion of 9–13 bp nucleotides (Figure 3a,b). These results indicated that ABE8e-EndoV could also accurately predict the deletion of small fragments, in addition to an efficient A-to-G substitution, and the principle of predicting precise deletion is similar to that of the previously reported APOBEC-Cas9 fusion-induced deletion systems [32]. ABE8e-EndoV can be applied to the study of A/T-rich elements. In addition, ABE8e-EndoV was fused with a broadly targeted Cas9 variant, SpGn, which helped achieve precise base editing and small-fragment deletions.

### 2.4. BER Pathway of Plant CGBE and ABE8e-EndoV

**Figure 4:**
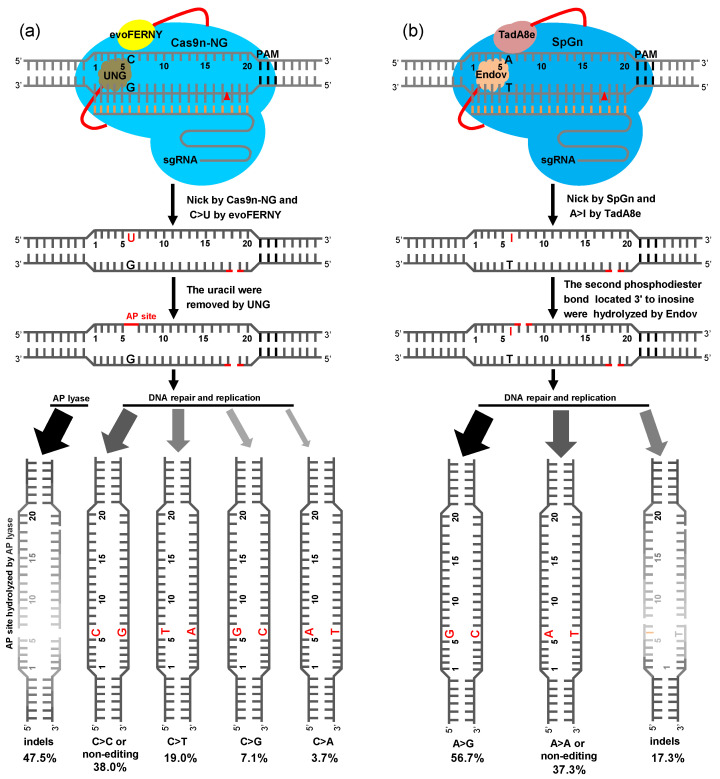
Plant CGBE- and ABE8e-EndoV-mediated excision repair pathway. (**a**) In the U base excision repair (BER) pathway of CGBE-rUNG and CGBE-hUNG, C bases in the editing window are deaminated by cytosine deaminase evoFERNY to U, U bases are excised by UNG to form an apurinic/apyrimidinic (AP) site, and then two repair pathways are initiated. In one of these pathways, the AP site is hydrolyzed by AP enzymes and activated by non-homologous end repair, leading to several indels, whereas in the other pathway, the AP site generates C-to-T, C-to-G, and C-to-A substitutions or restores original C after DNA repair and replication pathways. (**b**) The I base is the alternative BER (AER) pathway of ABE8e-EndoV. Bases are deaminated by adenine deaminase TadA8e to I, and the second phosphodiester bond located at the 3′-terminus of I bases is hydrolyzed by EndoV, resulting in A-to-G substitutions or the restoration of original A after DNA repair and replication. In addition, some indel mutations are generated after non-homologous end-joining repair. The gray area indicates the degree to which the indel frequency is generated.

We objectively evaluated the editing features of CGBE in rice. To more intuitively reveal the repair pathway of CGBE and ABE8e-EndoV, we summarize their base repair processes. In the U:G BER pathway of CGBE, C bases were deaminated by cytosine deaminase evoFERNY to U, U bases were excised by rUNG or hUNG to form an AP site, and then two repair pathways were initiated in the plants. The first pathway involves hydrolysis of the AP site by AP lyase and its subsequent activation by non-homologous end repair, resulting in several indels (47.5%). The position of indel mutations is mainly in the AP site within the editing window, most deletions extend to the 3′-terminus nick site of Cas9n-NG, and a small part extends to the 5′-terminus of the AP site (Figure 4a and Tables S1 and S2). In the second pathway, the AP site generates C-to-T (19.0%), C-to-G (7.1%), and C-to-A (3.7%) substitutions or the original C base after undergoing DNA repair and replication pathways (Figure 4a). Although 2xUGI was replaced by rUNG or hUNG, the base transversion efficiency of C-to-G and C-to-A was slightly improved, and that of C-to-T was significantly reduced. These results indicate that achieving base transversions without generating excessive indels in the editing window, such as in CGBE-TS1, CGBE-TS2, and CGBE-TS3, is challenging (Figure 1a and Figure S1).

In the editing of ABE8e-hAAG, only 4.3% deletion mutations were detected at the TS4 site (Figure 2b); hence, determining whether hAAG is less active or not active for the I:T BER pathway was difficult in this study. However, ABE8e-EndoV certainly played a key role in the I:T AER pathway; hence, only the AER pathway of ABE8e-EndoV is shown here. In the ABE8e-EndoV editing system, A bases were deaminated by adenine deaminase TadA8e to I, the I:T mismatch activated the AER pathway, and the second phosphodiester bond located at the 3′-terminus of I was hydrolyzed by EndoV. Finally, I-to-G (56.7%) or I-to-original A base substitutions were achieved through DNA repair and replication. In addition, indel mutations (17.3%) from DNA double-strand break and non-homologous end-joining repair were achieved. The region of indel mutations was mainly in the I base site within the editing window; a part of the deletion extended to the 3′-terminal nick site of SpG, and another part extended to the 5′-terminus of the I base (Figure 4b and Tables S3 and S4). The cleavage site of EndoV was at the second base of the 3′ of I, which did not generate an AP site with the breakage of the DNA strand. This could be the reason for the inefficiency of ABE8e-EndoV in generating the A-to-Y transversion.

## 3. Discussion

High-efficiency ABE and CBE base editing systems are vital in the study of gene function, genetic improvement, de novo domestication, and alternative splicing of mRNA [19,33]. However, base substitution types in ABE and CBE systems are limited and cannot meet the needs of various applications. Although CGBEs in mammalian cells effectively switch C-to-T substitutions to C-to-G transversions without causing more indel byproducts and effectively expand the applied range of base editors in animal genomes, in this study, both CGBE-rUNG and CGBE-hUNG produced indel byproducts, and the average C-to-G monoallelic editing efficiency, excluding indel mutations, was 7.1%; hence, CGBEs in plants should be further improved. Koblan et al. tested different UNG orthologs and found that UNG from Mycobacterium smegmatis (UdgX) moderately improved the purity of the C-to-G transversion product and the transversion efficiency, and the authors also reported that DNA polymerase D2 (POLD2) and RNA-binding motif protein X-linked (RBMX) would further improve the C-to-G editing efficiency [34]. Kurt et al. fused rAPOBEC1 (R33A) and *E. coli* eUNG to different positions of Cas9n, producing eUNG-rAPOBEC1-Cas9n (CGBE1) or removing the 2xUGI domain without adding the UNG domain, resulting in a high C-to-G editing efficiency [21]. However, whether these methods are effective for CGBE improvement in plants remains to be determined. Nevertheless, we speculated that the high activity of AP lyase in plants is one of the reasons for the significant indel byproducts. Inhibition of the AP lyase activity may effectively decrease the frequency of indels and increase the frequency of C-to-G transversion. We speculate that another reason for the significant indels is that consecutive C bases (≥2) within the editing window were synchronously deaminated to U bases in C-rich targets, and more than two consecutive U bases were removed by UNG to form consecutive AP sites. Multiple AP sites simultaneously hydrolyzed by AP lyase were more likely to result in high-frequency indels. However, in this case, we could not change the GC environment of the target sequence.

In eukaryotes, the low activity of BER enzymes for I:T mismatches results in the high purity of the ABE editing product. However, recent studies have reported that I:T mismatches will activate the BER and AER pathways [24,25,26,27,28]. In this study, ABE8e-EndoV, based on the AER pathway, achieved an A-to-indel breakthrough, which provides a basis for generating A-to-Y transversions in the next step. ABE8e-hAAG, based on the BER pathway, only detected 4.3% indels at TS4, suggesting that hAAG might play a role in I excision repair, but hAAG activity is possibly ineffective and requires further optimization. Using a protein-assisted evolution system is a common strategy to enhance the activity of hAAG and obtain superior AAG [1]. Another strategy is the screening of homologous AAGs from other species with high activity. Regarding the inefficiency of ABE8e-EndoV in producing A-to-Y transversion, we speculate that the cleavage site of EndoV at the second base of the 3′ of I, which does not generate an AP site with the DNA strand break, is the main reason. Based on the aforementioned analysis, we hypothesize that A-to-Y transversion could be achieved by screening high-activity AAG and constructing highly active ABE8e-AAG based on the BER pathway.

Overall, we systematically explored the editing performance of the CGBE system in rice, although we did not observe prominent C-to-G performance, as observed in previous studies on mammalian cells. We found some differences in the U base excise repair system between animals and plants, and our results provide a valuable reference for the improvement of CGBEs in plants. In the process of exploring the A-to-Y system, we found that ABE8e-EndoV can generate predictable precise small-fragment deletions of 9–13 bps, in addition to generating efficient A-to-G substitutions at the target sites. We conclude that ABE8e-EndoV can facilitate the study of *cis*-element and protein domains in plants.

## 4. Materials and Methods

### 4.1. Construction of Plant CGBE-rUNG, CGBE-hUNG, ABE8e-hAAG, and ABE8e-EndoV Vectors

To construct CGBE-rUNG and CGBE-hUNG vectors, we performed an NCBI BLAST search to obtain the amino acid sequences of UNG from humans (Sequence ID: 1AKZ_A) and rice (Sequence ID: XP_015634353.1) and then codon-optimized hUNG and rUNG amino acid sequences to nucleotide sequences based on the codon preference of rice, which were chemically synthesized by GeneCreate (Wuhan, China) (Sequence S1). PevoFERNY-Cas9n-NG preserved by our team was used as the basic vector for modification [2], and 2xUGI of evoFERNY-Cas9n-NG was replaced by rUNG and hUNG with overlapping PCR to obtain two fragments, namely, evoFERNY-Cas9n-NG-rUNG and evoFERNY-Cas9n-NG-hUNG. The two fragments were digested by *Pst* I and *BamH* I. Subsequently, pYLCRISPR/Cas9Pubi-H developed by our team was digested with *Pst* I and *BamH* I [35], and the 1300 binary vector backbone was extracted through agarose gel electrophoresis. Finally, the evoFERNY-Cas9n-NG-rUNG and evoFERNY-Cas9n-NG-hUNG fragments were ligated to the 1300 backbone by using T4 DNA ligase, and PevoFERNY-Cas9n-NG-rUNG (CGBE-rUNG) and PevoFERNY-Cas9n-NG-hUNG (CGBE-hUNG) vectors were obtained.

To construct ABE8e-hAAG and ABE8e-EndoV vectors, we also searched the protein sequences of hAAG from humans (Sequence ID: 1BNK_A) and EndoV from *E. coli* (Sequence ID: EGI38768.1) by using NCBI BLAST and then optimized the nucleic acid sequences according to the rice codon preferences and chemically synthesized them (Sequence S2). ABE8e-SpG preserved by our team was used as the basic vector for further modification [7]. hAAG and EndoV were respectively fused to the C-terminus of ABE8e-SpG by overlapping PCR. Finally, the ABE8e-SpGn-hAAG and ABE8e-SpGn-EndoV fragments were assembled into the 1300 binary vector through *Pst* I and *BamH* I digestion and T4 DNA ligation to construct ABE8e-SpGn-hAAG (ABE8e-hAAG) and ABE8e-SpGn-EndoV (ABE8e-EndoV).

### 4.2. Target Design and gRNA Expression Cassette Assembly

The five target sequences of CGBE-rUNG and CGBE-hUNG were derived from the coding DNA sequence regions of *LOC_Os02g58480* (*OsSUS6*), *LOC_Os01g69030* (*OsSPS1*), and *LOC_Os07g04230* (*Os07g0134700*), and then the CRISPR-GE web-based online tool [30] was used to analyze and select five C-rich targets (Figure 1b) [30]. The five targets of ABE8e-hAAG and ABE8e-EndoV were obtained from *LOC_Os01g68870* (*OsMSP1*), *LOC_Os05g06480* (*Chalk5*), and *LOC_Os01g10110* (*OsCKX2*) (Figure 2b), and CRISPR-GE was also used to analyze and select five A-rich targets. The sgRNA expression cassette was assembled into binary vectors by referring to the published multi-target assembly methods of CRISPR/Cas9 [35].

### 4.3. Genetic Transformation

All of the CGBE-rUNG, CGBE-hUNG, ABE8e-hAAG, and ABE8e-EndoV plasmids containing sgRNA expression cassettes were transformed into *Agrobacterium tumefaciens* (strain *EHA105*) through electroporation and then transformed into rice (*Oryza sativa*) (Nipponbare variety calli) by using the Agrobacterium-mediated method. T_0_ transgenic rice was obtained by callus induction and subculture, callus infection, co-culture, screening, differentiation of resistant callus, and rooting and transplantation of strong seedlings, according to previously reported [36].

### 4.4. On-Target and Off-Target Efficiency Analysis of T_0_ Rice

The genomic DNA of T_0_ plants was extracted using the SDS method. Target-specific primers with on-target and off-target efficiency for PCR and sequencing were designed using online CRISPR-GE tools. Our previous study results showed that selecting the on-target sequence difference with candidate off-target ≥3 bases can greatly reduce the off-target effects [29]. Candidate off-target sites with one to three base variations compared with the on-target sites of CGBE-rUNG, CGBE-hUNG, ABE8e-hAAG, and ABE8e-EndoV were analyzed and selected using the off-target subprogram of CRISPR-GE. To evaluate the on-target and off-target efficiencies, the target regions were amplified by PCR from the T_0_ plants by using site-specific primers, and barcode adapter primers and PCR product libraries were constructed. These PCR product libraries were sequenced using Illumina high-throughput deep sequencing (ANOROAD, Yiwu, China). The deep sequencing data were analyzed using Hi-TOM, which is an effective genome editing assessment tool [31].

## Supplementary Materials

The following supporting information can be downloaded at: https://www.mdpi.com/article/10.3390/ijms23147990/s1.
