# Supplementary material for: Exploring C-to-G and A-to-Y Base Editing in Rice by Using New Vector Tools
Source: Int J Mol Sci. 2022 Jul 20;23(14):7990. doi: 10.3390/ijms23147990 (PMC9318980; doi:10.3390/ijms23147990)
Supplement: Supplementary file 1 [file ijms-23-07990-s001.zip › ijms-1788402-supplementary.pdf]

Article

Exploring C-to-G and A-to-Y base editing in rice by using new vector tools

Figure S1. C-to-T, C-to-G and C-to-A substitution without indels efficiency of CGBE-rUNG-TS1~TS5 and CGBE-hUNG-TS1~TS5 during C_3-C_{12} window.

CGBE-rUNG-TS1

	G_3	C_4	C_5	C_6	T_7	C_8	C_9	T_{10}	C_{11}	C_{12}
C-T		3.7%	7.4%	3.7%		0	0		0	0
C-G		0	3.7%	0		0	0		0	0
C-A		0	0	0		0	0		0	0

CGBE-hUNG-TS1

	G_3	C_4	C_5	C_6	T_7	C_8	C_9	T_{10}	C_{11}	C_{12}
C-T		4.5%	4.5%	9.1%		0	0		0	0
C-G		0	0	4.5%		0	0		0	0
C-A		4.5%	0	0		0	0		0	0

CGBE-rUNG-TS2

	G_3	T_4	C_5	C_6	C_7	A_8	C_9	G_{10}	A_{11}	G_{12}
C-T			3.7%	0	3.7%		0			
C-G			0	0	0		0			
C-A			0	0	0		0			

CGBE-hUNG-TS2

	G_3	T_4	C_5	C_6	C_7	A_8	C_9	G_{10}	A_{11}	G_{12}
C-T			0	4.5%	13.6%		0			
C-G			0	0	0		0			
C-A			4.5%	9.1%	0		0			

CGBE-rUNG-TS3

	A_3	C_4	C_5	C_6	G_7	C_8	C_9	T_{10}	C_{11}	G_{12}
C-T		0	0	7.4%		0	0		0	
C-G		0	0	0		0	0		0	
C-A		0	0	0		0	0		0	

CGBE-hUNG-TS3

	A_3	C_4	C_5	C_6	G_7	C_8	C_9	T_{10}	C_{11}	G_{12}
C-T		4.5%	18.1%	18.1%		0	0		0	
C-G		0	0	4.5%		0	0		0	
C-A		0	4.5%	0		0	0		0	

CGBE-rUNG-TS4

	A_3	C_4	C_5	C_6	C_7	C_8	G_9	C_{10}	T_{11}	C_{12}
C-T		7.4%	29.6%	11.1%	18.5%	3.7%		0		0
C-G		0	0	3.7%	0	0		0		0
C-A		3.7%	7.4%	0	3.7%	0		0		0

CGBE-hUNG-TS4

	A_3	C_4	C_5	C_6	C_7	C_8	G_9	C_{10}	T_{11}	C_{12}
C-T			9.1%	9.1%	4.5%	9.1%		0		0
C-G			0	0	0	4.5%		0		0
C-A			0	0	0	0		0		0

CGBE-rUNG-TS6

	G_3	C_4	A_5	C_6	C_7	A_8	T_9	G_{10}	A_{11}	C_{12}
C-T		18.5%		18.5%	18.5%					0
C-G		3.7%		7.4%	11.1%					0
C-A		0		3.7%	0					0

CGBE-hUNG-TS6

	G_3	C_4	A_5	C_6	C_7	A_8	T_9	G_{10}	A_{11}	C_{12}
C-T		18.2%		13.6%	18.2%					0
C-G		4.5%		4.5%	18.2%					0
C-A		0		0	0					0

Figure S2. A-to-G, A-to-C and A-to-T substitution without indels efficiency of ABE8e-EndoV-TS1~TS5 and ABE8e-hAAG-TS1~TS5 during A_3-A_{12} window.

ABE8e-Endov-TS1

	C_3	T_4	C_5	A_6	A_7	C_8	T_9	T_{10}	C_{11}	A_{12}
A-G				36.7%	0					0
A-C				0	0					0
A-T				0	0					0

ABE8e-hAAG-TS1

	C_3	T_4	C_5	A_6	A_7	C_8	T_9	T_{10}	C_{11}	A_{12}
A-G				34.8%	0					0
A-C				0	0					0
A-T				0	0					0

ABE8e-Endov-TS2

	G_3	C_4	C_5	G_6	A_7	A_8	G_9	T_{10}	C_{11}	A_{12}
A-G					40.0%	26.7%				0
A-C					0	0				0
A-T					0	0				0

ABE8e-hAAG-TS2

	G_3	C_4	C_5	G_6	A_7	A_8	G_9	T_{10}	C_{11}	A_{12}
A-G					47.8%	26.1%				0
A-C					0	0				0
A-T					0	0				0

ABE8e-Endov-TS3

	G_3	A_4	A_5	A_6	A_7	A_8	G_9	C_{10}	C_{11}	G_{12}
A-G		3.3%	26.7%	60.0%	0	0				
A-C		0	0	0	0	0				
A-T		0	0	0	0	0				

ABE8e-hAAG-TS3

	G_3	A_4	A_5	A_6	A_7	A_8	G_9	C_{10}	C_{11}	G_{12}
A-G		8.7%	34.8%	39.1%	8.7%	0				
A-C		0	0	0	0	0				
A-T		0	0	0	0	0				

ABE8e-Endov-TS4

	A_3	A_4	G_5	A_6	A_7	G_8	A_9	C_{10}	G_{11}	A_{12}
A-G	10.0%	50.0%		73.3%	20.0%		0			0
A-C	0	0		0	0		0			0
A-T	0	0		0	0		0			0

ABE8e-hAAG-TS4

	A_3	A_4	G_5	A_6	A_7	G_8	A_9	C_{10}	G_{11}	A_{12}
A-G	13.0%	60.9%		82.6%	39.1%		0			0
A-C	0	0		0	0		0			0
A-T	0	0		0	0		0			0

ABE8e-Endov-TS5

	C_3	T_4	A_5	A_6	G_7	A_8	C_9	A_{10}	C_{11}	G_{12}
A-G			63.3%	23.3%		6.7%				
A-C			0	0		0		0		
A-T			0	0		0		0		

ABE8e-hAAG-TS5

	C_3	T_4	A_5	A_6	G_7	A_8	C_9	A_{10}	C_{11}	G_{12}
A-G			65.2%	47.8%		26.1%		0		
A-C			0	0		0		0		
A-T			0	0		0		0		

Figure S3. Off-target analyses of CGBE-rUNG, CGBE-hUNG, ABE8e-EndoV and ABE8e-hAAG.

(a)

Target site	Off-target sequence	CGBE-rUNG	CGBE-hUNG
OsSUS6-TS1	CTGCCCTCCTCCATCGGCAA**AGG**		
TS1-off1	CTGCCCTCCTCCTTCGCCAAAGT	0	0
TS1-off2	CTGCCCGCCTCCTTGGGCAACGG	0	0
TS1-off3	CGGCCGTCCTCCATCGGCACGGG	0	0
OsSUS6-TS2	CTGTCCCACGAGCTGGTACT**TGT**		
TS2-off1	CTGTCCCATCAGCTGGTTCTTGT	0	0
TS2-off2	CTCCACCACGAGCTGGTACTTGG	0	0
TS2-off3	CTGTCCCTAGAGCTGGGACTTGG	0	0
OsSPS1-TS3	GCACCCGCCTCGAGAACATG**TGC**		
TS3-off1	GCACCCAACTAGAGAACATGAGC	0	0
TS3-off2	GCACCCGCCTGGATCACATGCGC	0	0
TS3-off3	GCATCCGCCTCGAAGACATGAGG	0	0
OsSPS1-TS4	TGACCCCCGCTCGCCGTCGA**TGG**		
TS4-off1	TGACCCCTGGACGCCGTCGACGG	0	0
TS4-off2	TCCCCCCCGCTCGCCGTCGCCGG	0	4.5%
TS4-off3	TGCCCCGCGCTCGCCGTGGAAGG	0	0
Os07g0134700-TS5	TCGCACCATGACGCGCCGCA**GGT**		
TS5-off1	TCGCACCATGACTTACCGCACGG	0	0
TS5-off2	TCGCACCACGCCGCGCTGCAGGG	0	0
TS5-off3	TCGCACCATGACGCGCCGAATCA	0	0

(b)

Target site	Off-target sequence	ABE8e-Endov	ABE8e-hAAG
OsMSP1-TS1	GGCTCAACTTCAGTGGATGT**GGG**		
TS1-off1	GGCTCATCTTAAGTGGAGGTGGA	0	0
TS1-off2	GGCTCATCTTGAGTGGAGGTGGA	0	0
TS1-off3	GGCTCATCTTGAGTGGAGGTGGA	0	0
OsMSP1-TS2	AGGCCGAAGTCAGAGACCCT**CGG**		
TS2-off1	AGGCCGAAGTCGGAGACCCTGGC	3.3%	0
TS2-off2	AGGCCGAAGTCGGACACCCGCGG	0	0
TS2-off3	AGGCCGAAGTCGGAGACCTTGGC	0	0
TS2-off4	AGGCCGAAGTCGGAGACCTTGGC	0	0
Chalk5-TS3	GCGAAAAAGCCGCTACAAAC**GGC**		
TS3-off1	GGGAAAAAGCCGCTACTGACGGC	0	0
TS3-off2	GCGAAAAAGCCTCCACAAACTGA	0	0
TS3-off3	GCGAAAAAGCCGTGAGAAACGGT	0	0
Chalk5-TS4	AGAAGAAGACGATGAGCATT**AGG**		
TS4-off1	AGAAGATGACGATGAGCACTTGG	0	0
TS4-off2	AGAAGATGACGATGAGCATAGGT	0	0
TS4-off3	AGAAGAAGAAGAAGAACATTTGG	0	0

Table S1. The mutation of CGBE-rUNG in T_0 rice.

CGBE-rUNG-TS1

CGBE-rUNG	Ref:GACGCCTCACCTCACGCTGCCCTCCTCCATCGGCAA**AGG**CGCGCACTTGGTCTC		Allelic variation
TS1#07	A1: GACGCCTCACCTCACG-TGTT-TCCTCCATCGGCAA**AGG**CGCGCACTTGGTCTC	C>T,-2bp	Bia
	A2: GACGCCTCACCTCACGCTGTTTTCCTCCATCGGCAA**AGG**CGCGCACTTGGTCTC	C>T	
TS1#06	A1: GACGCCTCACCTCACGCTGCCCTCCTCCATCGGCAA**AGG**CGCGCACTTGGTCTC	WT	Het
	A2: GACGCCTCACCTCACGCTGC-TTCCTCCATCGGCAA**AGG**CGCGCACTTGGTCTC	C>T,-1bp	
TS1#03	A1: GACGCCTCACCTCACGCTGCCCTCCTCCATCGGCAA**AGG**CGCGCACTTGGTCTC	WT	Het
	A2: GACGCCTCACCTCACGCTG--CTCCTCCATCGGCAA**AGG**CGCGCACTTGGTCTC	-2bp	
TS1#13	A1: GACGCCTCACCTCACGCTGCCCTCCTCCATCGGCAA**AGG**CGCGCACTTGGTCTC	WT	Het
	A2: GACGCCTCACCTCACGTTG---TCCTCCATCGGCAA**AGG**CGCGCACTTGGTCTC	C>T,-3bp	
TS1#26	A1: GACGCCTCACCTCACGCTGCCCTCCTCCATCGGCAA**AGG**CGCGCACTTGGTCTC	WT	Het
	A2: GACGCCTCACCTCACG-------CCTCCATCGGCAA**AGG**CGCGCACTTGGTCTC	-7bp	
TS1#05	A1: GACGCCTCACCTCACGCTGCCCTCCTCCATCGGCAA**AGG**CGCGCACTTGGTCTC	WT	Het
	A2: GACGCCTCACCTCACGCTGCCCTC-------GGCAA**AGG**CGCGCACTTGGTCTC	-7bp	
TS1#15	A1: GACGCCTCACCTCACGCTGCCCTCCTCCATCGGCAA**AGG**CGCGCACTTGGTCTC	WT	Het
	A2: GACGCCTCACCTCACGCT----------ATCGGCAA**AGG**CGCGCACTTGGTCTC	-10bp	
TS1#11	A1: GACGCCTCACCTCACGCTGCC------------CAA**AGG**CGCGCACTTGGTCTC	-12bp	Bia
	A2: GACGCCTCACCTCACG----------CCATCGGCAA**AGG**CGCGCACTTGGTCTC	-10bp	
TS1#17	A1: GACGCCTCACCTCACGCTGCTGTCCTCCATCGGCAA**AGG**CGCGCACTTGGTCTC	C>T,C>G	Bia
	A2: GACGCCTCACCT---------------------CAA**AGG**CGCGCACTTGGTCTC	-21bp	
TS1#09	A1: GACGCCTCACC--------------------------**GG**CGCGCACTTGGTCTC	-26bp	Hom
	A2: GACGCCTCACC--------------------------**GG**CGCGCACTTGGTCTC	-26bp	
TS1#21	A1: GACGCCTCACCTCACGCTGCCCTCCTCCATCGGCAA**AGG**CGCGCACTTGGTCTC	WT	Het
	A2: GACGCCTCACCT-------------------------**GG**CGCGCACTTGGTCTC	-25bp	
TS1#12	A1: GATTTTGGCGCCCTGGACCTGTCGACGCCTCACCTCACGCTGCCCTCCTCCATCGGCAA**AGG**CGCGC	WT	Het
	A2: GATTTTGTG---TCGGCAA**AGG**CGCGC	-43bp	
TS1#02	A1: GACGCCTCACCTCACGCTGCCCTCCTCCACTCCTCCATCGGCAA**AGG**CGCGCACTTGGTCTC	+8bp	Bia
	A2: GACGCCTCACCTCACG-------CCTCCA--------TCGGCAA**AGG**CGCGCACTTGGTCTC	-7bp	

Bia, biallelic mutation; Het, heterozygous mutation; Hom, homozygous mutation.

CGBE-rUNG- TS2

CGBE-rUNG	Ref:TTTTATCCAACGGATCTGTCCCACGAGCTGGTACT**TGT**TTATCAGGCTGTGCA		Allelic variation
TS2#28	A1: TTTTATCCAACGGATCTGTCCCACGAGCTGGTACT**TGT**TTATCAGGCTGTGCA	WT	Het
	A2: TTTTATCCAACGGATCTGTTCTACGAGCTGGTACT**TGT**TTATCAGGCTGTGCA	C>T	
TS2#21	A1: TTTTATCCAACGGATCTGTCCCACGAGCTGGTACT**TGT**TTATCAGGCTGTGCA	WT	Het
	A2: TTTTATCCAACGGATCTG--ACACGAGCTGGTACT**TGT**TTATCAGGCTGTGCA	C>A,-2bp	
TS2#18	A1: TTTTATCCAACGGATCTGTCCCACGAGCTGGTACT**TGT**TTATCAGGCTGTGCA	WT	Het
	A2: TTTTATCCAACGGATCTGT--CACGAGCTGGTACT**TGT**TTATCAGGCTGTGCA	-2bp	
TS2#09	A1: TTTTATCCAACGGAT------CACGAGCTGGTACT**TGT**TTATCAGGCTGTGCA	-6bp	Hom
	A2: TTTTATCCAACGGAT------CACGAGCTGGTACT**TGT**TTATCAGGCTGTGCA	-6bp	
TS2#07	A1: TTTTATCCAACGGAT----------AGCTGGTACT**TGT**TTATCAGGCTGTGCA	-10bp	Bia

	A2: TTTTATCCAACGGATCTGTCCCA--AGCTGGTACT**TGT**TTATCAGGCTGTGCA -2bp	
TS2#08	A1: TTTTATCCAACGGATCTGTCCCACGAGCTGGTACT**TGT**TTATCAGGCTGTGCA WT	Het
	A2: TTTTATCCAACGGATCTGT----------CCTACT**TGT**TTATCAGGCTGTGCA -10bp	
TS2#12	A1: TTTTATCCAACGGATCTGTCCCACGAGCTGGTACT**TGT**TTATCAGGCTGTGCA WT	Het
	A2: TTTTATCCAACGGATCTGTC-----------TACT**TGT**TTATCAGGCTGTGCA -11bp	
TS2#02	A1: TTTTATCCAACGGATCTGT------------TACT**TGT**TTATCAGGCTGTGCA -12bp	Het
	A2: TTTTATCCAACGGATCTGTCCCACGAGCTGGTACT**TGT**TTATCAGGCTGTGCA WT	
TS2#15	A1: TTTTATCCAACGGATCTGTCCCACGAGCTGGTACT**TGT**TTATCAGGCTGTGCA WT	Het
	A2: TTTTATCCAACGGATCTGTC------------ACT**TGT**TTATCAGGCTGTGCA -12bp	
TS2#07	A1: TTTTATCCAACGGATCTGTCCCACGAGCTGGTACT**TGT**TTATCAGGCTGTGCA WT	Het
	A2: TTTTATCCAACGGATCTGT------------TACT**TGT**TTATCAGGCTGTGCA -12bp	
TS2#10	A1: TTTTATCCAACGGATCTGTCCCACGAGCTGGTACT**TGT**TTATCAGGCTGTGCA WT	Het
	A2: CTTTTATCCAACGGA------------------ACT**TGT**TTATCAGGCTGTGCA -18bp	
TS2#23	A1: TTTTATCCAACGGATCTGTCCCACGAGCTGGTACT**TGT**TTATCAGGCTGTGCA WT	Het
	A2: TTTTATCCAACGGATCT------------------**TGT**TTATCAGGCTGTGCA -18bp	
TS2#24	A1: TTTTATCCAACGGATCTGTCCCACGAGCTGGTACT**TGT**TTATCAGGCTGTGCA WT	Het
	A2: TTTTATCCAACGGATCT------------------**TGT**TTATCAGGCTGTGCA -18bp	
TS2#10	A1: TTTTATCCAA----------------------------------GGCTGTGCA -34bp	Het
	A2: TTTTATCCAACGGATCTGTCCCACGAGCTGGTACT**TGT**TTATCAGGCTGTGCA WT	
TS2#06	A1: CTCGGTCAGTCTGTCTTTTATCCAACGGATCTGTCCCACGAGCTGGTACT**TGT**TTATCAGGCTGT WT	Het
	A2: CTCGGT--------------------------------ACGAGCTGGTACT**TGT**TTATCAGGCTG -32bp	

CGBE-rUNG- TS3

CGBE-rUNG	Ref:ACGCCCGCGAGCGGAGCACCCGCCTCGAGAACATG**TGC**TGGCGCATCTGGCAC	Allelic variation
TS3#02	A1: ACGCCCGCGAGCGGAGCACCCGCCTCGAGAACATG**TGC**TGGCGCATCTGGCAC WT	Het
	A2: ACGCCCGCGAGCGGAGCACCTGCCTCGAGAACATG**TGC**TGGCGCATCTGGCAC C>T	
TS3#06	A1: ACGCCCGCGAGCGGAGCACCCGCCTCGAGAACATG**TGC**TGGCGCATCTGGCAC WT	Het
	A2: ACGCCCGCGAGCGGAGCACCCGCCTCGA---CATG**TGC**TGGCGCATCTGGCAC -3bp	
TS3#27	A1: ACGCCCGCGAGCGGAGCACCCGCCTCGAGAACATG**TGC**TGGCGCATCTGGCAC WT	Het
	A2: ACGCCCGCGAGCGGAGCAC--GCCTCGAGAACATG**TGC**TGGCGCATCTGGCAC C>T	
TS3#20	A1: ACGCCCGCGAGCGGAGCACCTGCCTCGAGAACATG**TGC**TGGCGCATCTGGCAC C>T	Bia
	A2: ACGCCCGCGAGCGGAGCAC----CTCGAGAACATG**TGC**TGGCGCATCTGGCAC -4bp	
TS3#13	A1: ACGCCCGCGAGCGGAGCACCCGCCTCGAGAACATG**TGC**TGGCGCATCTGGCAC WT	Het
	A2: ACGCCCGCGAA--------------CGAGAACATG**TGC**TGGCGCATCTGGCAC -14bp	
TS3#23	A1: ACGCCCGCGAGCGGAGCACCCGCCTCGAGAACATG**TGC**TGGCGCATCTGGCAC WT	Het
	A2: AC--------------------------------------GCGCATCTGGCAC -38bp	
TS3#24	A1: ACGCCCGCGAGCGGAGCACCCGCCTCGAGAACATG**TGC**TGGCGCATCTGGCAC WT	Het
	A2: AC--------------------------------------GCGCATCTGGCAC -38bp	
TS3#09	A1: GTCGTCGCCACCCGCAACGCCCGCGAGCGGAGCACCCGCCTCGAGAACATG**TGC**TGGCGCATCTG WT	Het
	A2: GTC---------------------------------------GAGAACATG**TGC**TGGCGCATCTG -39bp	

CGBE-rUNG-TS4

CGBE-rUNG	Ref:CGCGTCGTTCATGGATGACCCCCGCTCGCCGTCGA**TGG**ACAGGCGCAGCGAGA	Allelic variation

TS4#02	A1:	CGCGTCGTTCATGGATGAATTCTGCTCGCCGTCGA**TGG**ACAGGCGCAGCGAGA	C>T,C>A	Bia
	A2:	CGCGTCGTTCATGGATGACTGCCGCTCGCCGTCGA**TGG**ACAGGCGCAGCGAGA	C>T,C>G	
TS4#18	A1:	CGCGTCGTTCATGGATGACCCCCGCTCGCCGTCGA**TGG**ACAGGCGCAGCGAGA	WT	Het
	A2:	CGCGTCGTTCATGGATGACTCTCGCTCGCCGTCGA**TGG**ACAGGCGCAGCGAGA	C>T	
TS4#23	A1:	CGCGTCGTTCATGGATGACATCCGCTCGCCGTCGA**TGG**ACAGGCGCAGCGAGA	C>T,C>A	Bia
	A2:	CGCGTCGTTCATGGATGATTCTCGCTCGCCGTCGA**TGG**ACAGGCGCAGCGAGA	C>T	
TS4#24	A1:	CGCGTCGTTCATGGATGACATCCGCTCGCCGTCGA**TGG**ACAGGCGCAGCGAGA	C>T,C>A	Bia
	A2:	CGCGTCGTTCATGGATGATTCTCGCTCGCCGTCGA**TGG**ACAGGCGCAGCGAGA	C>T	
TS4#28	A1:	CGCGTCGTTCATGGATGACCCCCGCTCGCCGTCGA**TGG**ACAGGCGCAGCGAGA	WT	Het
	A2:	CGCGTCGTTCATGGATGACCCACGCTCGCCGTCGA**TGG**ACAGGCGCAGCGAGA	C>A	
TS4#01	A1:	CGCGTCGTTCATGGATGACCCCCGCTCGCCGTCGA**TGG**ACAGGCGCAGCGAGA	WT	Het
	A2:	CGCGTCGTTCATGGATGACTCCCGCTCGCCGTCGA**TGG**ACAGGCGCAGCGAGA	C>T	
TS4#26	A1:	CGCGTCGTTCATGGATGACCCCCGCTCGCCGTCGA**TGG**ACAGGCGCAGCGAGA	WT	Het
	A2:	CGCGTCGTTCATGGATGACTCCCGCTCGCCGTCGA**TGG**ACAGGCGCAGCGAGA	C>T	
TS4#05	A1:	CGCGTCGTTCATGGATGACCCCCGCTCGCCGTCGA**TGG**ACAGGCGCAGCGAGA	WT	Het
	A2:	CGCGTCGTTCATGGATGA---CCGCTCGCCGTCGA**TGG**ACAGGCGCAGCGAGA	-3bp	
TS4#14	A1:	CGCGTCGTTCATGGATGACCCCC---CGCCGTCGA**TGG**ACAGGCGCAGCGAGA	-3bp	Bia
	A2:	CGCGTCGTTCATGGATGACCCTCGCTCGCCGTCGA**TGG**ACAGGCGCAGCGAGA	C>T	
TS4#06	A1:	CGCGTCGTTCATGGATG-------CTCGCCGTCGA**TGG**ACAGGCGCAGCGAGA	-7bp	Bia
	A2:	CGCGTCGTTCATGGATGACCCCCGC--GTCGTTCA**TGG**ACAGGCGCAGCGAGA	-2bp,C>T	
TS4#09	A1:	CGCGTCGTTCATGGATGACCCCCGCTCGCCGTCGA**TGG**ACAGGCGCAGCGAGA	WT	Het
	A2:	CGCGTCGTTCATGGATGACC-------GCCGTCGA**TGG**ACAGGCGCAGCGAGA	-7bp	
TS4#12	A1:	CGCGTCGTTCATGGATGACCCCCGCTCGCCGTCGA**TGG**ACAGGCGCAGCGAGA	WT	Het
	A2:	CGCGTCGTTCATGGATGA-------TCGCCGTCGA**TGG**ACAGGCGCAGCGAGA	-7bp	
TS4#17	A1:	CGCGTCGTTCATGGATGAC----GCTCGCCGTCGA**TGG**ACAGGCGCAGCGAGA	-4bp	Bia
	A2:	CGCGTCGTTCATGGA------------GCCGTCGA**TGG**ACAGGCGCAGCGAGA	-12bp	
TS4#16	A1:	CGCGTCGTTCATGGATGA------------TGCGA**TGG**ACAGGCGCAGCGAGA	-12bp	Bia
	A2:	CGCGTCGTTCATGGATGA-CCCCGCTCGCCGTCGA**TGG**ACAGGCGCAGCGAGA	-1bp	
TS4#15	A1:	CGCGTCGTTCATGGATGACCCCCGCTCGCCGTCGA**TGG**ACAGGCGCAGCGAGA	WT	Het
	A2:	CGCGTCGTTCATGGATGACCCCCGCTCG-------------GGCGCAGCGAGA	-13bp	
TS4#20	A1:	CGCGTCGTTC-------------------CGTCGA**TGG**ACAGGCGCAGCGAGA	-19bp	Bia
	A2:	CGCGTCGTTCATGG----------CTCGCCGTCGA**TGG**ACAGGCGCAGCGAGA	-10bp	
TS4#27	A1:	CGCGTCGTTCATGGATGACCCCCGCTCGCCGTCGA**TGG**ACAGGCGCAGCGAGA	WT	Het
	A2:	C---GCGCAGCGAGA	-41bp	
TS4#10	A1:	AGTCCTGCGGGTCCGACGATGGCGCGTCGTTCATGGATGACCCCCGCTCGCCGTCGA**TGG**ACAGG	WT	Het
	A2:	AGTC---------------------------------------CCGCTCGCCGTCGA**TGG**ACAGG	-39bp	
TS4#13	A1:	CTGCGGGTC--------------------------------------CGTCGA**TGG**ACAGGCGCA	-38bp	Bia
	A2:	CTGCGGGTCCGACGATGGCGCGTCGTTCATGGATG-------CTCGCCGTCGA**TGG**ACAGGCGCA	-7bp	
TS4#22	A1:	GTCCGACGAT--CGAGA	-50bp	Bia
	A2:	GTCCGACGATGGCGCGTCGTTCATGGATGA------------TGCGA**TGG**ACAGGCGCAGCGAGA	-12bp	
TS4#21	A1:	TGGATGA--AG	-55bp	Het
	A2:	TGGATGACTCTCGCTCGCCGTCGA**TGG**ACAGGCGCAGCGAGAGGTCCTGGACGTCCATGAGCGAG	WT	
TS4#07	A1:	CGCGTCGTTCATGGATGACCCCCG------------------A**TGG**ACAGGCGCAGCGAGA	-10bp	Bia

	A2: CGCGTCGTTCATGGATGACCCCCGCTCGCCGTCTCGCCGTCGA**TGG**ACAGGCGCAGCGAGA +8bp		

CGBE-rUNG-TS5

CGBE-rUNG	Ref:GAGATCAATCAGCGGTCGCACCATGACGCGCCGCA**GGT**ACTGGTATTTTGGCC	Allelic variation
TS5#06	A1: GAGATCAATCAGCGGTCGTACGATGACGCGCCGCA**GGT**ACTGGTATTTTGGCC C>T,C>G<br>A2: GAGATCAATCAGCGGTCGCACTATGACGCGCCGCA**GGT**ACTGGTATTTTGGCC C>T	Bia
TS5#18	A1: GAGATCAATCAGCGGTCGCACCATGACGCGCCGCA**GGT**ACTGGTATTTTGGCC WT<br>A2: GAGATCAATCAGCGGTCGTAGCATGACGCGCCGCA**GGT**ACTGGTATTTTGGCC C>T,C>G	Het
TS5#03	A1: GAGATCAATCAGCGGTCGCACCATGACGCGCCGCA**GGT**ACTGGTATTTTGGCC WT<br>A2: GAGATCAATCAGCGGTCGTATCATGACGCGCCGCA**GGT**ACTGGTATTTTGGCC C>T	Het
TS5#04	A1: GAGATCAATCAGCGGTCGCATCATGACGCGCCGCA**GGT**ACTGGTATTTTGGCC WT<br>A2: GAGATCAATCAGCGGTCGTAGTATGACGCGCCGCA**GGT**ACTGGTATTTTGGCC C>T,C>G	Bia
TS5#08	A1: GAGATCAATCAGCGGTCGCACCATGACGCGCCGCA**GGT**ACTGGTATTTTGGCC WT<br>A2: GAGATCAATCAGCGGTCGCATTATGACGCGCCGCA**GGT**ACTGGTATTTTGGCC C>T	Het
TS5#11	A1: GAGATCAATCAGCGGTCGCACCATGACGCGCCGCA**GGT**ACTGGTATTTTGGCC WT<br>A2: GAGATCAATCAGCGGTCGCATCATGACGCGCCGCA**GGT**ACTGGTATTTTGGCC C>T	Het
TS5#26	A1: GAGATCAATCAGCGGTCGCACCATGACGCGCCGCA**GGT**ACTGGTATTTTGGCC WT<br>A2: GAGATCAATCAGCGGTCGCA-CATGACGCGCCGCA**GGT**ACTGGTATTTTGGCC -1bp	Het
TS5#07	A1: GAGATCAATCAGCGGTCG-AATATGACGCGCCGCA**GGT**ACTGGTATTTTGGCC -1bp,C>T<br>A2: GAGATCAATCAGCGGTCGCACGATGACGCGCTGCA**GGT**ACTGGTATTTTGGCC C>T,C>G	Bia
TS5#22	A1: GAGATCAATCAGCGGTCGCACCATGACGCGCCGCA**GGT**ACTGGTATTTTGGCC WT<br>A2: GAGATCAATCAGCGGTCGCA--ATGACGCGCCGCA**GGT**ACTGGTATTTTGGCC -2bp	Het
TS5#27	A1: GAGATCAATCAGCGGTCGCACCATGACGCGCCGCA**GGT**ACTGGTATTTTGGCC WT<br>A2: GAGATCAATCAGCGGTCGCAC--TGACGCGCCGCA**GGT**ACTGGTATTTTGGCC -2bp	Het
TS5#21	A1: GAGATCAATCAGCGGTCG---TATGACGCGCCGCA**GGT**ACTGGTATTTTGGCC C>T,-3bp<br>A2: GAGATCAATCAGCGGTCGCAACATGACGCGCCGCA**GGT**ACTGGTATTTTGGCC C>A	Bia
TS5#28	A1: GAGATCAATCAGCGGTCGTACGATGACGCGCCGCA**GGT**ACTGGTATTTTGGCC C>T,C>G<br>A2: GAGATCAATCAGCGGTCGC----TGACGCGCCGCA**GGT**ACTGGTATTTTGGCC -4bp	Bia
TS5#14	A1: GAGATCAATCAGCGGTCGCACCATGACGCGCCGCA**GGT**ACTGGTATTTTGGCC WT<br>A2: GAGATCAATCAGCGGTCGC---ATGACGCGCCGCA**GGT**ACTGGTATTTTGGCC -3bp	Het
TS5#09	A1: GAGATCAATCAGCGGTCGCACCATGACGCGCCGCA**GGT**ACTGGTATTTTGGCC WT<br>A2: GAGATCAATCAGCGGTC----------GCGCCGCA**GGT**ACTGGTATTTTGGCC -10bp	Het
TS5#15	A1: GAGATCAATCAGCGGTCGCACCATGACGCGCCGCA**GGT**ACTGGTATTTTGGCC WT<br>A2: GAGATCAATCAGCGGTCGCACC----------GCA**GGT**ACTGGTATTTTGGCC -10bp	Het
TS5#13	A1: GAGATCAATCAGCGGT---------------CGCA**GGT**ACTGGTATTTTGGCC -15bp<br>A2: GAGATCAAT------------CATGACGCGCCGCA**GGT**ACTGGTATTTTGGCC -12bp	Bia
TS5#19	A1: GAGATCAATCAGCGGTCGCACCATGACGCGCCGCA**GGT**ACTGGTATTTTGGCC WT<br>A2: GAGATCAATCAGCGGTC----------------CA**GGT**ACTGGTATTTTGGCC -16bp	Het
TS5#20	A1: GAGATCAATCAGCGGTCGCACCATGACGCGCCGCA**GGT**ACTGGTATTTTGGCC WT<br>A2: GAG----------------------ACGCGCCGCA**GGT**ACTGGTATTTTGGCC -22bp	Het
TS5#10	A1: GAGATCAATCAG------------------------**GT**ACTGGTATTTTGGCC -24bp<br>A2: GAGATCAATCAGCGGTCGGACTATGACGCGCCGCA**GGT**ACTGGTATTTTGGCC C>T,C>G	Bia
TS5#16	A1: GAGATCAATCAGCGGTCGCACCATGACGCGCCGCA**GGT**ACTGGTATTTTGGCC WT	Het

	A2: GAGATCAA-------------------------------- TGGTATTTTGGCC -32bp	
TS5#01	A1: GAGATCAATCAGCGGTCGC------------CGCA**GGT**ACTGGTATTTTGGCC -12bp	Bia
	A2: GAGATCAATCA---------------------------------------GCC -39bp	
TS5#02	A1: GAGATCAATCAGCGGTCGCACTATGACG---CGCCGCA**GGT**ACTGGTATTTTG C>T	Bia
	A2: GAGATCAATCAGCGGTCGCACCATGACGCGCCGCCGCA**GGT**ACTGGTATTTTG +3bp	
TS5#24	A1: GAGATCAATCAGCGGTCGCACCATGACG---CGCCGCA**GGT**ACTGGTATTTTG WT	Het
	A2: GAGATCAATCAGCGGTCGCACCATGACGCGCCGCCGCA**GGT**ACTGGTATTTTG +3bp	
TS5#23	A1: GAGATCAATCAGCGGTCGCACCATGA-----CGCGCCGCCGCA**GGT**ACTGGTA +3bp	Bia
	A2: GAGATCAATCAGCGGTCGCACCATGACGCGCCG---CGCCGCA**GGT**ACTGGTA +5bp	

Table S2. The mutation of CGBE-hUNG in T_0 rice.

CGBE-hUNG-TS1

CGBE-hUNG	Ref:ACGCCTCACCTCACGCTGCCCTCCTCCATCGGCAA**AGG**CGCGCACTTGGTCTC	Allelic variation
TS1#20	A1: ACGCCTCACCTCACGCTGCCCTCCTCCATCGGCAA**AGG**CGCGCACTTGGTCTC WT<br>A2: ACGCCTCACCTCACGGTGTCTTCCTCCATCGGCAA**AGG**CGCGCACTTGGTCTC C>T	Het
TS1#19	A1: ACGCCTCACCTCACGCTGCCCTCCTCCATCGGCAA**AGG**CGCGCACTTGGTCTC WT<br>A2: ACGCCTCACCTCACGTTGATCTCCTCCATCGGCAA**AGG**CGCGCACTTGGTCTC C>T,C>A	Het
TS1#03	A1: ACGCCTCACCTCACGCTGCCCTCCTCCATCGGCAA**AGG**CGCGCACTTGGTCTC WT<br>A2: ACGCCTCACCTCACGCTGCCGTCCTCCATCGGCAA**AGG**CGCGCACTTGGTCTC C>G	Het
TS1#14	A1: ACGCCTCACCTCACGCTGCCCTCCTCCATCGGCAA**AGG**CGCGCACTTGGTCTC WT<br>A2: ACGCCTCACCTCACGCTGCC-------ATCGGCAA**AGG**CGCGCACTTGGTCTC -7bp	Het
TS1#01	A1: ACGCCTCACCTCACGCTGCCCGA-------GGCAA**AGG**CGCGCACTTGGTCTC -7bp<br>A2: ACGCCTCACCTCACGCTGCCTTCCTCCATCGGCAA**AGG**CGCGCACTTGGTCTC C>T	Bia
TS1#06	A1: ACGCCTCACCTCACGCTGCCCTCCTCCATCGGCAA**AGG**CGCGCACTTGGTCTC WT<br>A2: ACGCCTCACCT---------CTCCTCCATCGGCAA**AGG**CGCGCACTTGGTCTC -9bp	Het
TS1#15	A1: ACGCCTCACCTCACGCTGCCGAT-------------GGCGCGCACTTGGTCTC -13bp<br>A2: ACGCCTCACCTCACGCTGCC--------------------CGCACTTGGTCTC -20bp	Bia
TS1#09	A1: ACGCCTCACCTCACGCTGCCCTCCTCCATCGGCAA**AGG**CGCGCACTTGGTCTC WT<br>A2: ACGCCTCACCTCAC-----------------GCAA**AGG**CGCGCACTTGGTCTC -17bp	Het
TS1#17	A1: ACGC-------------------CTCCATCGGCAA**AGG**CGCGCACTTGGTCTC -19bp<br>A2: ACGCCTCACCTCACGCTGCCCTCCTCCATCGGCAA**AGG**CGCGCACTTGGTCTC WT	Het
TS1#21	A1: ACGCCTCACCTCACGCTGCCCTCCTCCATCGGCAA**AGG**CGCGCACTTGGTCTC WT<br>A2: ACGCCTCACCT---------------------CAA**AGG**CGCGCACTTGGTCTC -21bp	Het
TS1#16	A1: TGTCACGCCTCACCTCACGCTGCCCTCCTCCATCGGCAA**AGG**CGCGCACTTGGTCTC WT<br>A2: TGT------------------------------CGGCAA**AGG**CGCGCACTTGGTCTC -30bp	Het
TS1#05	A1: CGCCTCACCTCACGCTGCCCTCCGACCCTCCATCGGCAA**AGG**CGCGCACTTGGTCTC +5bp<br>A2: CGCCTCACCTCACG--------------TCCATCGGCAA**AGG**CGCGCACTTGGTCTC -9bp	Bia
TS1#08	A1: TTG--TT -62bp<br>A2: TTGGCGCCCTGGACCTGTCGG----------------------CTCCATCGGCAA**AGG**CGCGCACTT -22bp	Bia
TS1#12	A1: CCTCACCTCACGCTGGCGATTAATTCGGAGTAGTTTGTGTCTTCCATCGGCAA**AGG**CGCGCACTTGG +21bp<br>A2: CCT---CGCGCACTTGG -32bp	Bia

Bia, biallelic mutation; Het, heterozygous mutation; Hom, homozygous mutation.

CGBE-hUNG-TS2

CGBE-hUNG	Ref:CTTTTATCCAACGGATCTGTCCCACGAGCTGGTACT**TGT**TTATCAGGCTGTG	Allelic variation
TS2#19	A1: CTTTTATCCAACGGATCTGTCCCACGAGCTGGTACT**TGT**TTATCAGGCTGTG WT<br>A2: CTTTTATCCAACGGATCTGTCATACGAGCTGGTACT**TGT**TTATCAGGCTGTG C>T,C>A	Het
TS2#17	A1: CTTTTATCCAACGGATCTGTCCCACGAGCTGGTACT**TGT**TTATCAGGCTGTG WT<br>A2: CTTTTATCCAACGGATCTGT---ACGAGCTGGTACT**TGT**TTATCAGGCTGTG -3bp	Het
TS2#08	A1: CTTTTATCCAACGGATCTGTCCCACGAGCTGGTACT**TGT**TTATCAGGCTGTG WT<br>A2: CTTTTATCCAACGGAT-------ACGAGCTGGTACT**TGT**TTATCAGGCTGTG -7bp	Het
TS2#15	A1: CTTTTATCCAACGGATCTGTC------------ACT**TGT**TTATCAGGCTGTG -12bp<br>A2: CTTTTATCCAACGGATCTGTAATACGAGCTGGTACT**TGT**TTATCAGGCTGTG C>T,C>A	Bia

TS2#05	A1: CTTTTATCCAACGGATCTGTCCCACGAGCTGGTACT**TGT**TTATCAGGCTGTG WT<br>A2: CTTTTATCCAACGGATCT-------------GTACT**TGT**TTATCAGGCTGTG -13bp	Het
TS2#01	A1: CTTTTATCCAACGGATCTGT------------TACT**TGT**TTATCAGGCTGTG -12bp<br>A2: CTTTTATCCAACGGATC----------------ACT**TGT**TTATCAGGCTGTG -15bp	Bia
TS2#06	A1: CTTTTATCCAACGGATCT----------------TT**TGT**TTATCAGGCTGTG -16bp<br>A2: CTTTTATCCAACGGATCT-------------GTACT**TGT**TTATCAGGCTGTG -13bp	Bia
TS2#16	A1: TATCCAACGGATCTGTCC-------------------------------------TCTATC -37bp<br>A2: TATCCAACGGATCTGTCTTACGAGCTGGTACT**TGT**TTATCAGGCTGTGCATCTTGTTTATC C>T	Bia
TS2#04	A1: CAGTCTGTCCTTTTATCCAACGGATCTGTCCCACGAGCTGGTACT**TGT**TTATCAGGCTGTG WT<br>A2: CAGTCTGTC---------------------------------------TTATCAGGCTGTG -39bp	Het

CGBE-hUNG-TS3

CGBE-hUNG	Ref:ACGCCCGCGAGCGGA<u>GCACCCGCCTCGAGAACATG</u>**TGC**TGGCGCATCTGGCAC	Allelic variation
TS3#12	A1: ACGCCCGCGAGCGGAGCACCCGCCTCGAGAACATG**TGC**TGGCGCATCTGGCAC WT<br>A2: ACGCCCGCGAGCGGAGCACCGGCCTCGAGAACATG**TGC**TGGCGCATCTGGCAC C>G	Het
TS3#16	A1: ACGCCCGCGAGCGGAGCACTTGCCTCGAGAACATG**TGC**TGGCGCATCTGGCAC C>T<br>A2: ACGCCCGCGAGCGGAGCACTTGCCTCGAGAACATG**TGC**TGGCGCATCTGGCAC C>T	Hom
TS3#01	A1: ACGCCCGCGAGCGGAGCACCCGCCTCGAGAACATG**TGC**TGGCGCATCTGGCAC WT<br>A2: ACGCCCGCGAGCGGAGCATTTGCCTCGAGAACATG**TGC**TGGCGCATCTGGCAC C>T	Het
TS3#21	A1: ACGCCCGCGAGCGGAGCACCCGCCTCGAGAACATG**TGC**TGGCGCATCTGGCAC WT<br>A2: ACGCCCGCGAGCGGAGCACACGCCTCGAGAACATG**TGC**TGGCGCATCTGGCAC C>A	Het
TS3#18	A1: ACGCCCGCGAGCGGAGCACCCGCCTCGAGAACATG**TGC**TGGCGCATCTGGCAC WT<br>A2: ACGCCCGCGAGCGGAGCACTTGCCTCGAGAACATG**TGC**TGGCGCATCTGGCAC C>T	Het
TS3#17	A1: ACGCCCGCGAGCGGAGCACCCGCCTCGAGAACATG**TGC**TGGCGCATCTGGCAC WT<br>A2: ACGCCCGCGAGCGGAGCACC------GAGAACATG**TGC**TGGCGCATCTGGCAC -6bp	Het
TS3#08	A1: ACGCCCGCGAGCGGAGCAC-------------ATG**TGC**TGGCGCATCTGGCAC -13bp<br>A2: ACGCCCGCGA------------CCTCGAGAACATG**TGC**TGGCGCATCTGGCAC -12bp	Bia
TS3#5	A1: ACGCCCGCGAGCGGAGCACCCGCCTCGAGAACATG**TGC**TGGCGCATCTGGCAC WT<br>A2: ACGCCCGCGAGCGGAGCAA-------------ATG**TGC**TGGCGCATCTGGCAC -13bp	Het
TS3#15	A1: CCCGCGAGCGGAGCACC------------ATG**TGC**TGGCGCATCTGGCAC -12bp<br>A2: CCCGCGAGCGGAGCACCCGCCGCCTCGAGAACATG**TGC**TGGCGCATCTGGCAC +3bp	Bia
TS3#19	A1: CGTCGCCACCCGCAACGCCCGCGAGCGGAGCACCCGCCTCGAGAACATG**TGC**TGGCGCATCTGGCAC WT<br>A2: CGT-------------------------------TGCCTCGAGAACATG**TGC**TGGCGCATCTGGCAC -31bp	Het
TS3#04	A1: CG---TCGAGAACATG**TGC**TGGCGCATCTGGCAC -49bp<br>A2: CGTGGATCAAGGTCGTCGCCACCCGCAACGCCCGCGAGCGGAGCACTTGCCTCGAGAACATG**TGC**TGGCGCATCTGGCAC C>T	Bia

CGBE-hUNG-TS4

CGBE-hUNG	Ref:CGCGTCGTTCATGGA<u>TGACCCCCGCTCGCCGTCGA</u>**TGG**ACAGGCGCAGCGAGA	Allelic variation
TS4#6	A1: CGCGTCGTTCATGGATGA---CCGCTCGCCGTCGA**TGG**ACAGGCGCAGCGAGA -3bp<br>A2: CGCGTCGTTCATGGATGACCCCGGCTCGCCGTCGA**TGG**ACAGGCGCAGCGAGA C>G	Bia
TS4#18	A1: CGCGTCGTTCATGGATGACCCCCGCTCGCCGTCGA**TGG**ACAGGCGCAGCGAGA WT<br>A2: CGCGTCGTTCATGGATGA-CCCCGCTCGCCGTCGA**TGG**ACAGGCGCAGCGAGA -1bp	Het

TS4#17	A1: CGTCGTTCATGGATGACCCCCGCTCGCGCCGTCGA**TGG**ACAGGCGCAGCGAGA +2bp<br>A2: CGTCGTTCATGGATGACTCCCGCT--CGCCGTCGA**TGG**ACAGGCGCAGCGAGA C>T	Bia
TS4#16	A1: CGCGTCGTTCATGGATGACCCCCGCTCGCCGTCGA**TGG**ACAGGCGCAGCGAGA WT<br>A2: CGCGTCGTTCATGGATGA--CCCGCTCGCCGTCGA**TGG**ACAGGCGCAGCGAGA -2bp	Het
TS4#05	A1: GTCGTTCATGGATGACCCCCGCTGCTCGCCGTCGA**TGG**ACAGGCGCAGCGAGA +3bp<br>A2: GTCGTTCATGGATGACCTCC---GCTCGCCGTCGA**TGG**ACAGGCGCAGCGAGA C>T	Bia
TS4#14	A1: TCGTTCATGGATGACCCCCGCTCG----CCGTCGA**TGG**ACAGGCGCAGCGAGA WT<br>A2: TCGTTCATGGATGACCCCCGCTCGCCGTCCGTCGA**TGG**ACAGGCGCAGCGAGA +3bp	Het
TS4#20	A1: CGCGTCGTTCATGGATGACCCCCGCTCGCCGTCGA**TGG**ACAGGCGCAGCGAGA WT<br>A2: CGCGTCGTTCATGGA------ACGCTCGCCGTCGA**TGG**ACAGGCGCAGCGAGA -6bp,C>A	Het
TS4#21	A1: CGCGTCGTTCATGGA------ACGCTCGCCGTCGA**TGG**ACAGGCGCAGCGAGA -6bp,C>A<br>A2: CGCGTCGTTCATGGATGACC-------GCCGTCGA**TGG**ACAGGCGCAGCGAGA -7bp	Bia
TS4#15	A1: CGCGTCGTTCATGGATG-------CTCGCCGTCGA**TGG**ACAGGCGCAGCGAGA -7bp<br>A2: CGCGTCGTTCATGGATGA------------GTCGA**TGG**ACAGGCGCAGCGAGA -12bp	Bia
TS4#19	A1: CGCGTCGTTCATGGATGAC--------------GA**TGG**ACAGGCGCAGCGAGA -14bp<br>A2: CGCGTCGTTCATGGATGAC---TGCTCGCCGTCGA**TGG**ACAGGCGCAGCGAGA -3bp,C>T	Bia
TS4#01	A1: CGCGTCGTTCATGGA------------------GA**TGG**ACAGGCGCAGCGAGA -18bp<br>A2: CGCGTCGTTCATGGATGA---CCGCTCGCCGTCGA**TGG**ACAGGCGCAGCGAGA -3bp	Bia
TS4#10	A1: CGCGTCGTTCATGGATGACCCTTGCTCGCCGTCGA**TGG**ACAGGCGCAGCGAGA CC>TT<br>A2: CGCGTCGTTC-------------------CGTCGA**TGG**ACAGGCGCAGCGAGA -19bp	Bia
TS4#09	A1: CGCGTCG----------------------CGTCGA**TGG**ACAGGCGCAGCGAGA -22bp<br>A2: CGCGTCGTTCATGGATGACC----------GTCGA**TGG**ACAGGCGCAGCGAGA -10bp	Bia
TS4#11	A1: CGCGTCGTTCATGG------------------------ACAGGCGCAGCGAGA -24bp<br>A2: CGCGTCGTTCATGGATGACCCCCGCTC-----CGA**TGG**ACAGGCGCAGCGAGA -5bp	Bia
TS4#12	A1: CGCGTCGTTCATGG------------------------ACAGGCGCAGCGAGA -24bp<br>A2: CGCGTCGTTCATGGATGACCCC-----------GA**TGG**ACAGGCGCAGCGAGA -11bp	Bia
TS4#08	A1: CGACGATGGCGCGTCGTTCATGGATGAC------------TCGA**TGG**ACAGGCGCAGCGAGA -12bp<br>A2: CGACGATG--------------------------------------GACAGGCGCAGCGAGA -38bp	Bia
TS4#04	A1: CTCTGCACCGAGTCCTGCGGGTCCGACGATGGCGCGTCGTTCATGGATGACTTCTGCTCGCCG**TCG**A C>T<br>A2: CTCTGC---TCGCCG**TCG**A -51bp	Bia

CGBE-hUNG-TS5

CGBE-hUNG	Ref:GAGATCAATCAGCGGTCGCACCATGACGCGCCGCA**GGT**ACTGGTATTTTGGCC	Allelic variation
TS6#13	A1: GAGATCAATCAGCGGTCGCACCATGACGCGCCGCA**GGT**ACTGGTATTTTGGCC WT<br>A2: GAGATCAATCAGCGGTCGTACGATGACGCGCCGCA**GGT**ACTGGTATTTTGGCC C>T,C>G	Het
TS6#08	A1: GAGATCAATCAGCGGTCGCACTATGACGCGCCGCA**GGT**ACTGGTATTTTGGCC C>T<br>A2: GAGATCAATCAGCGGTCGCAGCATGACGCGCCGCA**GGT**ACTGGTATTTTGGCC C>G	Bia
TS6#09	A1: GAGATCAATCAGCGGTCGTATGATGACGCGCCGCA**GGT**ACTGGTATTTTGGCC C>T,C>G<br>A2: GAGATCAATCAGCGGTCGCACGATGACGCGCCGCA**GGT**ACTGGTATTTTGGCC C>G	Bia
TS6#11	A1: GAGATCAATCAGCGGTCGCACCATGACGCGCCGCA**GGT**ACTGGTATTTTGGCC WT<br>A2: GAGATCAATCAGCGGTCGCATCATGACGCGCCGCA**GGT**ACTGGTATTTTGGCC C>T	Het
TS6#19	A1: GAGATCAATCAGCGGTCGTACCATGACGCGCCGCA**GGT**ACTGGTATTTTGGCC C>T<br>A2: GAGATCAATCAGCGGTCGCACCATGACGCGCCGCA**GGT**ACTGGTATTTTGGCC WT	Het

TS6#16	A1: GAGATCAATCAGCGGTCGCACCATGACGCGCCCGCA**GGT**ACTGGTATTTTGGCC +1bp<br>A2: GAGATCAATCAGCGGTCGGACTATGACGCG-CCGCA**GGT**ACTGGTATTTTGGCC C>G,C>T	Bia
TS6#06	A1: GAGATCAATCAGCGGTCGTATCATGACGCGCCGCA**GGT**ACTGGTATTTTGGCC C>T<br>A2: GAGATCAATCAGCGGTC----TATGACGCGCCGCA**GGT**ACTGGTATTTTGGCC -4bp	Bia
TS6#14	A1: GAGATCAATCAGCGGTCGCACCATGACG---CGCA**GGT**ACTGGTATTTTGGCC -3bp<br>A2: GAGATCAATCAGCGGTCGCACCATGACG---CGCA**GGT**ACTGGTATTTTGGCC -3bp	Hom
TS6#21	A1: GAGATCAATCAGCGGTCGCAC------GCGCCGCA**GGT**ACTGGTATTTTGGCC -6bp<br>A2: GAGATCAATCAGCGGTCGCACCATGA-----CGCA**GGT**ACTGGTATTTTGGCC -5bp	Bia
TS6#04	A1: GAGATCAATCAGCGGTCGCACCA----------CA**GGT**ACTGGTATTTTGGCC -10bp<br>A2: GAGATCAATCAGCGGTCGCACGATGACGCGCCGCA**GGT**ACTGGTATTTTGGCC C>G	Bia
TS6#15	A1: GAGATCAATCAGCGGTCGCACC----------GCA**GGT**ACTGGTATTTTGGCC -10bp<br>A2: GAGATCAATCAGCGGTCGCACGATGACGCGCCGCA**GGT**ACTGGTATTTTGGCC C>G	Bia
TS6#05	A1: GAGATCAATCAGCGGT---------------CGCA**GGT**ACTGGTATTTTGGCC -15bp<br>A2: GAGATCAATCAGCGGTCGCACTATGACGCGCCGCA**GGT**ACTGGTATTTTGGCC C>T	Bia
TS6#17	A1: GAGATCAATCAGCGGTCGCACTATGACGCGCCGCA**GGT**ACTGGTATTTTGGCC C>T<br>A2: GAGATCAATCAGCGG---------------------GTACTGGTATTTTGGCC -21bp	Bia
TS6#10	A1: GAGATCAATCAGCGGTCGCACCATGACGCGCCGCA**GGT**ACTGGTATTTTGGCC WT<br>A2: GAGATCAAT---------------------GTGCA**GGT**ACTGGTATTTTGGCC -21bp	Het
TS6#01	A1: GAG---------------------------------GTACTGGTATTTTGGCC -33bp<br>A2: GAGATCAATCAGCGGTCG---CATGACGCGCCGCA**GGT**ACTGGTATTTTGGCC -3bp	Bia
TS6#12	A1: CAATCAGCGGT--------------------CGCA**GGT**ACTGGTATTTTGGCC -15bp<br>A2: CAATCAGCGGTCGCACCATGACGCGCCGCGCCGCA**GGT**ACTGGTATTTTGGCC +5bp	Bia
TS6#20	A1: TCAATCAGCGGTCGCACCA------GCCGCA**GGT**ACTGGTATTTTGGCCTTAGAACA -6bp<br>A2: TCAATCAGCGGTCGCACCATGACGC----------------------------AACA -28bp	Bia

Table S3. The mutation of ABE8e-EndoV in T_0 rice.

ABE8e-EndoV-TS1

ABE8e-Endov	Ref:TCCAGTCACTTGTTCGGCTCAACTTCAGTGGATGTGGGTTTTCCGGAGAGCTT	Allelic variation
TS1#7	A1: TCCAGTCACTTGTTCGGCTCAACTTCAGTGGATGTGGGTTTTCCGGAGAGCTT WT<br>A2: TCCAGTCACTTGTTCGGCTCGACTTCAGTGGATGTGGGTTTTCCGGAGAGCTT A>G	Het
TS1#15	A1: TCCAGTCACTTGTTCGGCTCAACTTCAGTGGATGTGGGTTTTCCGGAGAGCTT WT<br>A2: TCCAGTCACTTGTTCGGCTCGACTTCAGTGGATGTGGGTTTTCCGGAGAGCTT A>G	Het
TS1#10	A1: TCCAGTCACTTGTTCGGCTCAACTTCAGTGGATGTGGGTTTTCCGGAGAGCTT WT<br>A2: TCCAGTCACTTGTTCGGCTCGACTTCAGTGGATGTGGGTTTTCCGGAGAGCTT A>G	Het
TS1#11	A1: TCCAGTCACTTGTTCGGCTCAACTTCAGTGGATGTGGGTTTTCCGGAGAGCTT WT<br>A2: TCCAGTCACTTGTTCGGCTCGACTTCAGTGGATGTGGGTTTTCCGGAGAGCTT A>G	Het
TS1#16	A1: TCCAGTCACTTGTTCGGCTCAACTTCAGTGGATGTGGGTTTTCCGGAGAGCTT WT<br>A2: TCCAGTCACTTGTTCGGCTCGACTTCAGTGGATGTGGGTTTTCCGGAGAGCTT A>G	Het
TS1#18	A1: TCCAGTCACTTGTTCGGCTCAACTTCAGTGGATGTGGGTTTTCCGGAGAGCTT WT<br>A2: TCCAGTCACTTGTTCGGCTCGACTTCAGTGGATGTGGGTTTTCCGGAGAGCTT A>G	Het
TS1#19	A1: TCCAGTCACTTGTTCGGCTCGACTTCAGTGGATGTGGGTTTTCCGGAGAGCTT A>G<br>A2: TCCAGTCACTTGTTCGGCTCGACTTCAGTGGATGTGGGTTTTCCGGAGAGCTT A>G	Hom
TS1#21	A1: TCCAGTCACTTGTTCGGCTCAACTTCAGTGGATGTGGGTTTTCCGGAGAGCTT WT<br>A2: TCCAGTCACTTGTTCGGCTCGACTTCAGTGGATGTGGGTTTTCCGGAGAGCTT A>G	Het
TS1#22	A1: TCCAGTCACTTGTTCGGCTCAACTTCAGTGGATGTGGGTTTTCCGGAGAGCTT WT<br>A2: TCCAGTCACTTGTTCGGCTCGACTTCAGTGGATGTGGGTTTTCCGGAGAGCTT A>G	Het
TS1#26	A1: TCCAGTCACTTGTTCGGCTCAACTTCAGTGGATGTGGGTTTTCCGGAGAGCTT WT<br>A2: TCCAGTCACTTGTTCGGCTCAAC---AGTGGATGTGGGTTTTCCGGAGAGCTT -3bp	Het
TS1#08	A1: TCCAGTCACTTGTTCGGCTCAAC---------AGTGGGTTTTCCGGAGAGCTT -9bP<br>A2: TCCAGTCACTTGTTCGGCTCGACTTCAGTGGATGTGGGTTTTCCGGAGAGCTT A>G	Bia
TS1#05	A1:TTCCAGTCACTTGTTCGGCTCGACTTCAGTGGATGTGGGTTTTCCGGAGAGCTT A>G<br>A2:T-----------------------TTCAGTGGATGTGGGTTTTCCGGAGAGCTT -23bp	Bia

Bia, biallelic mutation; Het, heterozygous mutation; Hom, homozygous mutation.

ABE8e-EndoV-TS2

ABE8e-Endov	Ref:CTGATGATCCTTGCAAGGCCGAAGTCAGAGACCCTCGGCTCGAAGTTCTCATC	Allelic variation
TS2#02	A1: CTGATGATCCTTGCAAGGCCGGAGTCAGAGACCCTCGGCTCGAAGTTCTCATC A>G<br>A2: CTGATGATCCTTGCAAGGCCGAGGTCAGAGACCCTCGGCTCGAAGTTCTCATC A>G	Bia
TS2#05	A1: CTGATGATCCTTGCAAGGCCGGAGTCAGAGACCCTCGGCTCGAAGTTCTCATC A>G<br>A2: CTGATGATCCTTGCAAGGCCGGGGTCAGAGACCCTCGGCTCGAAGTTCTCATC A>G	Bia
TS2#06	A1: CTGATGATCCTTGCAAGGCCGGAGTCAGAGACCCTCGGCTCGAAGTTCTCATC A>G<br>A2: CTGATGATCCTTGCAAGGCCGGGGTCAGAGACCCTCGGCTCGAAGTTCTCATC A>G	Bia
TS2#09	A1: CTGATGATCCTTGCAAGGCCGGAGTCAGAGACCCTCGGCTCGAAGTTCTCATC A>G<br>A2: CTGATGATCCTTGCAAGGCCGGGGTCAGAGACCCTCGGCTCGAAGTTCTCATC A>G	Bia
TS2#10	A1: CTGATGATCCTTGCAAGGCCGAAGTCAGAGACCCTCGGCTCGAAGTTCTCATC WT<br>A2: CTGATGATCCTTGCAAGGCCGGAGTCAGAGACCCTCGGCTCGAAGTTCTCATC A>G	Het
TS2#12	A1: CTGATGATCCTTGCAAGGCCGAAGTCAGAGACCCTCGGCTCGAAGTTCTCATC WT<br>A2: CTGATGATCCTTGCAAGGCCGGAGTCAGAGACCCTCGGCTCGAAGTTCTCATC A>G	Het

TS2#13	A1: CTGATGATCCTTGCAAGGCCGAAGTCAGAGACCCT**CGG**CTCGAAGTTCTCATC WT<br>A2: CTGATGATCCTTGCAAGGCCGGAGTCAGAGACCCT**CGG**CTCGAAGTTCTCATC A>G	Het
TS2#14	A1: CTGATGATCCTTGCAAGGCCGAAGTCAGAGACCCT**CGG**CTCGAAGTTCTCATC WT<br>A2: CTGATGATCCTTGCAAGGCCGGAGTCAGAGACCCT**CGG**CTCGAAGTTCTCATC A>G	Het
TS2#20	A1: CTGATGATCCTTGCAAGGCCGAAGTCAGAGACCCT**CGG**CTCGAAGTTCTCATC WT<br>A2: CTGATGATCCTTGCAAGGCCGGAGTCAGAGACCCT**CGG**CTCGAAGTTCTCATC A>G	Het
TS2#24	A1: CTGATGATCCTTGCAAGGCCGGAGTCAGAGACCCT**CGG**CTCGAAGTTCTCATC A>G<br>A2: CTGATGATCCTTGCAAGGCCGGGGTCAGAGACCCT**CGG**CTCGAAGTTCTCATC A>G	Bia
TS2#25	A1: CTGATGATCCTTGCAAGGCCGAAGTCAGAGACCCT**CGG**CTCGAAGTTCTCATC WT<br>A2: CTGATGATCCTTGCAAGGCCGGGGTCAGAGACCCT**CGG**CTCGAAGTTCTCATC A>G	Het
TS2#28	A1: CTGATGATCCTTGCAAGGCCGGAGTCAGAGACCCT**CGG**CTCGAAGTTCTCATC A>G<br>A2: CTGATGATCCTTGCAAGGCCGAGGTCAGAGACCCT**CGG**CTCGAAGTTCTCATC A>G	Bia
TS2#17	A1: CTGATGATCCTTGCAAGGCCGG-GTCAGAGACCCT**CGG**CTCGAAGTTCTCATC -1bp,A>G<br>A2: CTGATGATCCTTGCAAGGCCGAAGTCAGAGACCCT**CGG**CTCGAAGTTCTCATC WT	Het
TS2#23	A1: CTGATGATCCTTGCAAGGCCGG-GTCAGAGACCCT**CGG**CTCGAAGTTCTCATC -1bp,A>G<br>A2: CTGATGATCCTTGCAAGGCCGAGGTCAGAGACCCT**CGG**CTCGAAGTTCTCATC A>G	Bia
TS2#29	A1: CTGATGATCCTTGCAAGGCCGAAGTCAGAGACCCT**CGG**CTCGAAGTTCTCATC WT<br>A2: CTGATGATCCTTGCAAGGCCGG-GTCAGAGACCCT**CGG**CTCGAAGTTCTCATC -1bp,A>G	Het
TS2#30	A1: CTGATGATCCTTGCAAGGCCGAAGTCAGAGACCCT**CGG**CTCGAAGTTCTCATC WT<br>A2: CTGATGATCCTTGCAAGGCCGG-GTCAGAGACCCT**CGG**CTCGAAGTTCTCATC -1bp,A>G	Het
TS2#08	A1: CTGATGATCCTTGCAAGGCTGAGGGTCAGAGACCCT**CGG**CTCGAAGTTCTCAT +1bp,A>G<br>A2: CTGATGATCCTTGCAAGGC-CGAGGTCAGAGACCCT**CGG**CTCGAAGTTCTCAT A>G	Bia
TS2#16	A1: ATGATCCTTGCAAGGCCGAG-------GTCAGAGACCCT**CGG**CTCGAAGTTCTCATC A>G<br>A2: ATGATCCTTGCAAGGCCGAAGTCTCTTGTCAGAGACCCT**CGG**CTCGAAGTTCTCATC +7bp	Bia

ABE8e-EndoV-TS3

ABE8e-Endov	Ref:TCACCAAGAAAAGTAGCGAAAAAGCCGCTACAAAC**GGC**ACCGTCTAGAATCCT	Allelic variation
TS3#01	A1: TCACCAAGAAAAGTAGCGAGGAAGCCGCTACAAAC**GGC**ACCGTCTAGAATCCT A>G<br>A2: TCACCAAGAAAAGTAGCGAGGAAGCCGCTACAAAC**GGC**ACCGTCTAGAATCCT A>G	Hom
TS3#04	A1: TCACCAAGAAAAGTAGCGAAAAAGCCGCTACAAAC**GGC**ACCGTCTAGAATCCT WT<br>A2: TCACCAAGAAAAGTAGCGAAGAAGCCGCTACAAAC**GGC**ACCGTCTAGAATCCT A>G	Het
TS3#07	A1: TCACCAAGAAAAGTAGCGAAAAAGCCGCTACAAAC**GGC**ACCGTCTAGAATCCT WT<br>A2: TCACCAAGAAAAGTAGCGAAGAAGCCGCTACAAAC**GGC**ACCGTCTAGAATCCT A>G	Het
TS3#08	A1: TCACCAAGAAAAGTAGCGAGGAAGCCGCTACAAAC**GGC**ACCGTCTAGAATCCT A>G<br>A2: TCACCAAGAAAAGTAGCGAAGAAGCCGCTACAAAC**GGC**ACCGTCTAGAATCCT A>G	Bia
TS3#10	A1: TCACCAAGAAAAGTAGCGAAAAAGCCGCTACAAAC**GGC**ACCGTCTAGAATCCT WT<br>A2: TCACCAAGAAAAGTAGCGAAGAAGCCGCTACAAAC**GGC**ACCGTCTAGAATCCT A>G	Het
TS3#12	A1: TCACCAAGAAAAGTAGCGAAAAAGCCGCTACAAAC**GGC**ACCGTCTAGAATCCT WT<br>A2: TCACCAAGAAAAGTAGCGAGGAAGCCGCTACAAAC**GGC**ACCGTCTAGAATCCT A>G	Het
TS3#15	A1: TCACCAAGAAAAGTAGCGAAAAAGCCGCTACAAAC**GGC**ACCGTCTAGAATCCT WT<br>A2: TCACCAAGAAAAGTAGCGGAGAAGCCGCTACAAAC**GGC**ACCGTCTAGAATCCT A>G	Het
TS3#16	A1: TCACCAAGAAAAGTAGCGAAAAAGCCGCTACAAAC**GGC**ACCGTCTAGAATCCT WT<br>A2: TCACCAAGAAAAGTAGCGAAGAAGCCGCTACAAAC**GGC**ACCGTCTAGAATCCT A>G	Het

TS3#13	A1: TCACCAAGAAAAGTAGCGAAAAAGCCGCTACAAACGGCACCGTCTAGAATCCT WT	Het
	A2: TCACCAAGAAAAGTAGCGAAGAAGCCGCTACAAACGGCACCGTCTAGAATCCT A>G	
TS3#17	A1: TCACCAAGAAAAGTAGCGAAAAAGCCGCTACAAACGGCACCGTCTAGAATCCT WT	Het
	A2: TCACCAAGAAAAGTAGCGAGGAAGCCGCTACAAACGGCACCGTCTAGAATCCT A>G	
TS3#11	A1: TCACCAAGAAAAGTAGCGAGGAAGCCGCTACAAACGGCACCGTCTAGAATCCT A>G	Het
	A2: TCACCAAGAAAAGTAGCGAAAAAGCCGCTACAAACGGCACCGTCTAGAATCCT WT	
TS3#24	A1: TCACCAAGAAAAGTAGCGAAAAAGCCGCTACAAACGGCACCGTCTAGAATCCT WT	Het
	A2: TCACCAAGAAAAGTAGCGAAGAAGCCGCTACAAACGGCACCGTCTAGAATCCT A>G	
TS3#28	A1: TCACCAAGAAAAGTAGCGAAAAAGCCGCTACAAACGGCACCGTCTAGAATCCT WT	Het
	A2: TCACCAAGAAAAGTAGCGAAGAAGCCGCTACAAACGGCACCGTCTAGAATCCT A>G	
TS3#29	A1: TCACCAAGAAAAGTAGCGAAAAAGCCGCTACAAACGGCACCGTCTAGAATCCT WT	Het
	A2: TCACCAAGAAAAGTAGCGAGGAAGCCGCTACAAACGGCACCGTCTAGAATCCT A>G	
TS3#30	A1: TCACCAAGAAAAGTAGCGAAAAAGCCGCTACAAACGGCACCGTCTAGAATCCT WT	Het
	A2: TCACCAAGAAAAGTAGCGAGGAAGCCGCTACAAACGGCACCGTCTAGAATCCT A>G	
TS3#05	A1: TCACCAAGAAAAGTAGCGAAAAAGCCGCTACAAACGGCACCGTCTAGAATCCT WT	Het
	A2: TCACCAAGAAAAGTAGCGA-GGAGCCGCTACAAACGGCACCGTCTAGAATCCT -1bp	
TS3#03	A1: TCACCAAGAAAAGTAGCGAGG--------TTTGGACGCACCGTCTAGAATCCT -8bp	Bia
	A2: TCACCAAGAAAAGTAGCGAAGAAGCCGCTACAAACGGCACCGTCTAGAATCCT A>G	
TS3#23	A1: TCACCAAGAAAAGTAGCGAGGAAGCCGCTACAAACGGCACCGTCTAGAATCCT A>G	Bia
	A2: TCACCAAGAAAAGTAGCGA-----------GGAACGGCACCGTCTAGAATCCT -11bp	

ABE8e-EndoV-TS4

ABE8e-Endov	Ref:ACGCATGGATAAACAAGAAGAAGACGATGAGCATTAGGGTTCCTTAACCAGTT	Allelic variation
TS4#01	A1: ACGCATGGATAAACAAGAAGGAGACGATGAGCATTAGGGTTCCTTAACCAGTT A>G	Bia
	A2: ACGCATGGATAAACAAGAGGGAGACGATGAGCATTAGGGTTCCTTAACCAGTT A>G	
TS4#03	A1: ACGCATGGATAAACAAGAAGGAGACGATGAGCATTAGGGTTCCTTAACCAGTT A>G	Bia
	A2: ACGCATGGATAAACAAGAGGGGGACGATGAGCATTAGGGTTCCTTAACCAGTT A>G	
TS4#05	A1: ACGCATGGATAAACAAGAAGGAGACGATGAGCATTAGGGTTCCTTAACCAGTT A>G	Hom
	A2: ACGCATGGATAAACAAGAAGGAGACGATGAGCATTAGGGTTCCTTAACCAGTT A>G	
TS4#06	A1: ACGCATGGATAAACAAGAAGGAGACGATGAGCATTAGGGTTCCTTAACCAGTT A>G	Bia
	A2: ACGCATGGATAAACAAGAGGGAGACGATGAGCATTAGGGTTCCTTAACCAGTT A>G	
TS4#08	A1: ACGCATGGATAAACAAGAGGGAGACGATGAGCATTAGGGTTCCTTAACCAGTT A>G	Bia
	A2: ACGCATGGATAAACAAGGAGAAGACGATGAGCATTAGGGTTCCTTAACCAGTT A>G	
TS4#10	A1: ACGCATGGATAAACAAGAGGAAGACGATGAGCATTAGGGTTCCTTAACCAGTT A>G	Bia
	A2: ACGCATGGATAAACAAGAAGGAGACGATGAGCATTAGGGTTCCTTAACCAGTT A>G	
TS4#13	A1: ACGCATGGATAAACAAGAAGGAGACGATGAGCATTAGGGTTCCTTAACCAGTT A>G	Bia
	A2: ACGCATGGATAAACAAGGAGGAGACGATGAGCATTAGGGTTCCTTAACCAGTT A>G	
TS4#14	A1: ACGCATGGATAAACAAGAAGGAGACGATGAGCATTAGGGTTCCTTAACCAGTT A>G	Bia
	A2: ACGCATGGATAAACAAGAGGGAGACGATGAGCATTAGGGTTCCTTAACCAGTT A>G	
TS4#15	A1: ACGCATGGATAAACAAGAAGAAGACGATGAGCATTAGGGTTCCTTAACCAGTT WT	Het
	A2: ACGCATGGATAAACAAGAGGAAGACGATGAGCATTAGGGTTCCTTAACCAGTT A>G	
TS4#17	A1: ACGCATGGATAAACAAGAGGAAGACGATGAGCATTAGGGTTCCTTAACCAGTT A>G	Bia
	A2: ACGCATGGATAAACAAGAGGGAGACGATGAGCATTAGGGTTCCTTAACCAGTT A>G	

TS4#18	A1:	ACGCATGGATAAACAAGAAGAAGACGATGAGCATTAGGGTTCCTTAACCAGTT	WT	Het
	A2:	ACGCATGGATAAACAAGAGGAAGACGATGAGCATTAGGGTTCCTTAACCAGTT	A>G	
TS4#11	A1:	ACGCATGGATAAACAAGGGGGAGACGATGAGCATTAGGGTTCCTTAACCAGTT	A>G	Bia
	A2:	ACGCATGGATAAACAAGAAGAGGACGATGAGCATTAGGGTTCCTTAACCAGTT	A>G	
TS4#20	A1:	ACGCATGGATAAACAAGAAGGAGACGATGAGCATTAGGGTTCCTTAACCAGTT	A>G	Bia
	A2:	ACGCATGGATAAACAAGAGGGAGACGATGAGCATTAGGGTTCCTTAACCAGTT	A>G	
TS4#23	A1:	ACGCATGGATAAACAAGAGGGAGACGATGAGCATTAGGGTTCCTTAACCAGTT	A>G	Bia
	A2:	ACGCATGGATAAACAAGAGGGGGACGATGAGCATTAGGGTTCCTTAACCAGTT	A>G	
TS4#24	A1:	ACGCATGGATAAACAAGAAGGAGACGATGAGCATTAGGGTTCCTTAACCAGTT	A>G	Bia
	A2:	ACGCATGGATAAACAAGAGGGAGACGATGAGCATTAGGGTTCCTTAACCAGTT	A>G	
TS4#25	A1:	ACGCATGGATAAACAAGAAGAAGACGATGAGCATTAGGGTTCCTTAACCAGTT	WT	Het
	A2:	ACGCATGGATAAACAAGAAGGAGACGATGAGCATTAGGGTTCCTTAACCAGTT	A>G	
TS4#27	A1:	ACGCATGGATAAACAAGAAGAA--CGATGAGCATTAGGGTTCCTTAACCAGTT	-2bp	Bia
	A2:	ACGCATGGATAAACAAGAAGGAGACGATGAGCATTAGGGTTCCTTAACCAGTT	A>G	
TS4#29	A1:	ACGCATGGATAAACAAGAAGGAGACGATGAGCATTAGGGTTCCTTAACCAGTT	A>G	Bia
	A2:	ACGCATGGATAAACAAGAGG---ACGATGAGCATTAGGGTTCCTTAACCAGTT	A>G,-3bp	
TS4#28	A1:	ACGCATGGATAAACAAGAGGGGGACGATGAGCATTAGGGTTCCTTAACCAGTT	A>G	Bia
	A2:	ACGCATGGATAAACAAGAAA---ACGATGAGCATTAGGGTTCCTTAACCAGTT	A>G,-3bp	
TS4#04	A1:	ACGCATGGATAAACAAGAAGA-----------ATTAGGGTTCCTTAACCAGTT	-11bp	Bia
	A2:	ACGCATGGATAAACAAGAGGGAGACGATGAGCATTAGGGTTCCTTAACCAGTT	A>G	
TS4#07	A1:	ACGCATGGATAAACAAGAAGA-----------ATTAGGGTTCCTTAACCAGTT	-11bp	Bia
	A2:	ACGCATGGATAAACAAGAAGGGGACGATGAGCATTAGGGTTCCTTAACCAGTT	A>G	
TS4#09	A1:	ACGCATGGATAAACAAGAAGA-----------ATTAGGGTTCCTTAACCAGTT	-11bp	Bia
	A2:	ACGCATGGATAAACAAGAAGAAGACGATGAGCATTAGGGTTCCTTAACCAGTT	A>G	
TS4#12	A1:	ACGCATGGATAAACAAGAAGG-----------ATTAGGGTTCCTTAACCAGTT	A>G,-11bp	Bia
	A2:	ACGCATGGATAAACAAGAAGGAGACGATGAGCATTAGGGTTCCTTAACCAGTT	A>G	
TS4#16	A1:	ACGCATGGATAAACAAGAAGA-----------ATTAGGGTTCCTTAACCAGTT	-11bp	Bia
	A2:	ACGCATGGATAAACAAGAAGGGGACGATGAGCATTAGGGTTCCTTAACCAGTT	A>G	
TS4#02	A1:	ACGCATGGATAAACAAGAAGG-----------ATTAGGGTTCCTTAACCAGTT	-11bp	Bia
	A2:	ACGCATGGATAAACAAGGG---GACGATGAGCATTAGGGTTCCTTAACCAGTT	A>G,-3bp	

ABE8e-EndoV-TS5

ABE8e-Endov	Ref:	TAGATCCATCACATCTACTAAGACACGCGCCGCCATGATCGAGGTAATTAAGG		Allelic variation
TS5#01	A1:	TAGATCCATCACATCTACTAAGGCACGCGCCGCCATGATCGAGGTAATTAAGG	A>G	Bia
	A2:	TAGATCCATCACATCTACTGAGACACGCGCCGCCATGATCGAGGTAATTAAGG	A>G	
TS5#04	A1:	TAGATCCATCACATCTACTAAGACACGCGCCGCCATGATCGAGGTAATTAAGG	WT	Het
	A2:	TAGATCCATCACATCTACTGAGACACGCGCCGCCATGATCGAGGTAATTAAGG	A>G	
TS5#06	A1:	TAGATCCATCACATCTGCTAAGACACGCGCCGCCATGATCGAGGTAATTAAGG	A>G	Bia
	A2:	TAGATCCATCACATCTACTGAGACACGCGCCGCCATGATCGAGGTAATTAAGG	A>G	
TS5#07	A1:	TAGATCCATCACATCTACTAAGACACGCGCCGCCATGATCGAGGTAATTAAGG	WT	Het
	A2:	TAGATCCATCACATCTACTGAGACACGCGCCGCCATGATCGAGGTAATTAAGG	A>G	
TS5#08	A1:	TAGATCCATCACATCTACTGGGACACGCGCCGCCATGATCGAGGTAATTAAGG	A>G	Hom

	A2: TAGATCCATCACATCTACTGGGACACGCGCCGCCA**TGA**TCGAGGTAATTAAGG A>G	
TS5#12	A1: TAGATCCATCACATCTACTGAGACACGCGCCGCCA**TGA**TCGAGGTAATTAAGG A>G<br>A2: TAGATCCATCACATCTACTGGGACACGCGCCGCCA**TGA**TCGAGGTAATTAAGG A>G	Bia
TS5#15	A1: TAGATCCATCACATCTACTAAGACACGCGCCGCCA**TGA**TCGAGGTAATTAAGG WT<br>A2: TAGATCCATCACATCTACTGGGACACGCGCCGCCA**TGA**TCGAGGTAATTAAGG A>G	Het
TS5#16	A1: TAGATCCATCACATCTACTAAGACACGCGCCGCCA**TGA**TCGAGGTAATTAAGG WT<br>A2: TAGATCCATCACATCTACTGAGACACGCGCCGCCA**TGA**TCGAGGTAATTAAGG A>G	Het
TS5#05	A1: TAGATCCATCACATCTACTGAGACACGCGCCGCCA**TGA**TCGAGGTAATTAAGG<br>A2: TAGATCCATCACATCTACTGGGACACGCGCCGCCA**TGA**TCGAGGTAATTAAGG A>G	Bia
TS5#17	A1: TAGATCCATCACATCTACTAAGACACGCGCCGCCA**TGA**TCGAGGTAATTAAGG WT<br>A2: TAGATCCATCACATCTACTGAGACACGCGCCGCCA**TGA**TCGAGGTAATTAAGG A>G	Het
TS5#18	A1: TAGATCCATCACATCTACTAAGACACGCGCCGCCA**TGA**TCGAGGTAATTAAGG WT<br>A2: TAGATCCATCACATCTACTGAGACACGCGCCGCCA**TGA**TCGAGGTAATTAAGG A>G	Het
TS5#11	A1: TAGATCCATCACATCTACTAAGACACGCGCCGCCA**TGA**TCGAGGTAATTAAGG WT<br>A2: TAGATCCATCACATCTACTGAGACACGCGCCGCCA**TGA**TCGAGGTAATTAAGG A>G	Het
TS5#20	A1: TAGATCCATCACATCTACTAAGACACGCGCCGCCA**TGA**TCGAGGTAATTAAGG WT<br>A2: TAGATCCATCACATCTACTGAGACACGCGCCGCCA**TGA**TCGAGGTAATTAAGG A>G	Het
TS5#24	A1: TAGATCCATCACATCTACTAAGACACGCGCCGCCA**TGA**TCGAGGTAATTAAGG WT<br>A2: TAGATCCATCACATCTACTGAGACACGCGCCGCCA**TGA**TCGAGGTAATTAAGG A>G	Het
TS5#29	A1: TAGATCCATCACATCTACTGAGACACGCGCCGCCA**TGA**TCGAGGTAATTAAGG A>G<br>A2: TAGATCCATCACATCTACTGAGGCACGCGCCGCCA**TGA**TCGAGGTAATTAAGG A>G	Bia
TS5#23	A1: TAGATCCATCACATCTACTGAGACACGCGCCGCCA**TGA**TCGAGGTAATTAAGG A>G<br>A2: TAGATCCATCACATCTACTGGGACACGCGCCGCCA**TGA**TCGAGGTAATTAAGG A>G	Bia
TS5#10	A1: TAGATCCATCACATCTACTGAGACACGCGCCGCCA**TGA**TCGAGGTAATTAAGG A>G<br>A2: TAGATCCATCACATCTACT----CACGCGCCGCCA**TGA**TCGAGGTAATTAAGG -4bp	Bia
TS5#19	A1: TAGATCCATCACATCTACT------CGCGCCGCCA**TGA**TCGAGGTAATTAAGG -6bp<br>A2: TAGATCCATCACATCTACTGGGACACGCGCCGCCA**TGA**TCGAGGTAATTAAGG A>G	Bia
TS5#13	A1: TAGATCCATCACATCTACTGGGACACGCGCCGCCA**TGA**TCGAGGTAATTAAGG A>G<br>A2: TAGATCCATCACATCTACT-------------CCA**TGA**TCGAGGTAATTAAGG -13bp	Bia
TS5#09	A1: TAGATCCATCACATCTACTAAGACACGCGCCGCCA**TGA**TCGAGGTAATTAAGG WT<br>A2: TAGATCCATCACATCTA---------------CCA**TGA**TCGAGGTAATTAAGG -15bp	Het
TS5#30	A1: TAGATCCATCACATCTACT---------------------GAGGTAATTAAGG -21bp<br>A2: TAGATCCATCACATCTACTGAGACACGCGCCGCCA**TGA**TCGAGGTAATTAAGG A>G	Bia

Table S4. The mutation of ABE8e-hAAG in T_0 rice.

ABE8e-hAAG-TS1

ABE8e-hAAG	Ref:TCCAGTCACTTGTTCGGCTCAACTTCAGTGGATGTGGGTTTTCCGGAGAGCTT	Allelic variation
TS1#01	A1: TCCAGTCACTTGTTCGGCTCAACTTCAGTGGATGTGGGTTTTCCGGAGAGCTT WT<br>A2: TCCAGTCACTTGTTCGGCTCGACTTCAGTGGATGTGGGTTTTCCGGAGAGCTT A>G	Het
TS1#10	A1: TCCAGTCACTTGTTCGGCTCAACTTCAGTGGATGTGGGTTTTCCGGAGAGCTT WT<br>A2: TCCAGTCACTTGTTCGGCTCGACTTCAGTGGATGTGGGTTTTCCGGAGAGCTT A>G	Het
TS1#13	A1: TCCAGTCACTTGTTCGGCTCAACTTCAGTGGATGTGGGTTTTCCGGAGAGCTT WT<br>A2: TCCAGTCACTTGTTCGGCTCGACTTCAGTGGATGTGGGTTTTCCGGAGAGCTT A>G	Het
TS1#14	A1: TCCAGTCACTTGTTCGGCTCAACTTCAGTGGATGTGGGTTTTCCGGAGAGCTT WT<br>A2: TCCAGTCACTTGTTCGGCTCGACTTCAGTGGATGTGGGTTTTCCGGAGAGCTT A>G	Het
TS1#18	A1: TCCAGTCACTTGTTCGGCTCAACTTCAGTGGATGTGGGTTTTCCGGAGAGCTT WT<br>A2: TCCAGTCACTTGTTCGGCTCGACTTCAGTGGATGTGGGTTTTCCGGAGAGCTT A>G	Het
TS1#19	A1: TCCAGTCACTTGTTCGGCTCAACTTCAGTGGATGTGGGTTTTCCGGAGAGCTT WT<br>A2: TCCAGTCACTTGTTCGGCTCGACTTCAGTGGATGTGGGTTTTCCGGAGAGCTT A>G	Het
TS1#21	A1: TCCAGTCACTTGTTCGGCTCAACTTCAGTGGATGTGGGTTTTCCGGAGAGCTT WT<br>A2: TCCAGTCACTTGTTCGGCTCGACTTCAGTGGATGTGGGTTTTCCGGAGAGCTT A>G	Het
TS1#23	A1: TCCAGTCACTTGTTCGGCTCAACTTCAGTGGATGTGGGTTTTCCGGAGAGCTT WT<br>A2: TCCAGTCACTTGTTCGGCTCGACTTCAGTGGATGTGGGTTTTCCGGAGAGCTT A>G	Het

Bia, biallelic mutation; Het, heterozygous mutation; Hom, homozygous mutation.

ABE8e-hAAG-TS2

ABE8e-hAAG	Ref:CTGATGATCCTTGCAAGGCCGAAGTCAGAGACCCTCGGCTCGAAGTTCTCATC	Allelic variation
TS2#01	A1: CTGATGATCCTTGCAAGGCCGAAGTCAGAGACCCTCGGCTCGAAGTTCTCATC WT<br>A2: CTGATGATCCTTGCAAGGCCGGGGTCAGAGACCCTCGGCTCGAAGTTCTCATC A>G	Het
TS2#05	A1: CTGATGATCCTTGCAAGGCCGAAGTCAGAGACCCTCGGCTCGAAGTTCTCATC WT<br>A2: CTGATGATCCTTGCAAGGCCGGAGTCAGAGACCCTCGGCTCGAAGTTCTCATC A>G	Het
TS2#08	A1: CTGATGATCCTTGCAAGGCCGAAGTCAGAGACCCTCGGCTCGAAGTTCTCATC WT<br>A2: CTGATGATCCTTGCAAGGCCGGAGTCAGAGACCCTCGGCTCGAAGTTCTCATC A>G	Het
TS2#10	A1: CTGATGATCCTTGCAAGGCCGGAGTCAGAGACCCTCGGCTCGAAGTTCTCATC A>G<br>A2: CTGATGATCCTTGCAAGGCCGGGGTCAGAGACCCTCGGCTCGAAGTTCTCATC A>G	Bia
TS2#13	A1: CTGATGATCCTTGCAAGGCCGGGGTCAGAGACCCTCGGCTCGAAGTTCTCATC A>G<br>A2: CTGATGATCCTTGCAAGGCCGGAGTCAGAGACCCTCGGCTCGAAGTTCTCATC A>G	Bia
TS2#14	A1: CTGATGATCCTTGCAAGGCCGAGGTCAGAGACCCTCGGCTCGAAGTTCTCATC A>G<br>A2: CTGATGATCCTTGCAAGGCCGGGGTCAGAGACCCTCGGCTCGAAGTTCTCATC A>G	Bia
TS2#16	A1: CTGATGATCCTTGCAAGGCCGAAGTCAGAGACCCTCGGCTCGAAGTTCTCATC WT<br>A2: CTGATGATCCTTGCAAGGCCGGAGTCAGAGACCCTCGGCTCGAAGTTCTCATC A>G	Het
TS2#18	A1: CTGATGATCCTTGCAAGGCCGAAGTCAGAGACCCTCGGCTCGAAGTTCTCATC WT<br>A2: CTGATGATCCTTGCAAGGCCGGAGTCAGAGACCCTCGGCTCGAAGTTCTCATC A>G	Het
TS2#19	A1: CTGATGATCCTTGCAAGGCCGGGGTCAGAGACCCTCGGCTCGAAGTTCTCATC A>G<br>A2: CTGATGATCCTTGCAAGGCCGGAGTCAGAGACCCTCGGCTCGAAGTTCTCATC A>G	Bia
TS2#20	A1: CTGATGATCCTTGCAAGGCCGAGGTCAGAGACCCTCGGCTCGAAGTTCTCATC A>G<br>A2: CTGATGATCCTTGCAAGGCCGGGGTCAGAGACCCTCGGCTCGAAGTTCTCATC A>G	Bia

TS2#23	A1: CTGATGATCCTTGCAAGGCCGAAGTCAGAGACCCT**CGG**CTCGAAGTTCTCATC WT	Het
	A2: CTGATGATCCTTGCAAGGCCGGAGTCAGAGACCCT**CGG**CTCGAAGTTCTCATC A>G	

ABE8e-hAAG-TS3

ABE8e-hAAG	Ref:TCACCAAGAAAAGTAGCGAAAAAGCCGCTACAAAC**GGC**ACCGTCTAGAATCCT	Allelic variation
TS3#1	A1: TCACCAAGAAAAGTAGCGAAAAAGCCGCTACAAAC**GGC**ACCGTCTAGAATCCT WT	Het
	A2: TCACCAAGAAAAGTAGCGGGGAAGCCGCTACAAAC**GGC**ACCGTCTAGAATCCT A>G	
TS3#10	A1: TCACCAAGAAAAGTAGCGAAAAAGCCGCTACAAAC**GGC**ACCGTCTAGAATCCT WT	Het
	A2: TCACCAAGAAAAGTAGCGAGGGAGCCGCTACAAAC**GGC**ACCGTCTAGAATCCT A>G	
TS3#13	A1: TCACCAAGAAAAGTAGCGAAAAAGCCGCTACAAAC**GGC**ACCGTCTAGAATCCT WT	Het
	A2: TCACCAAGAAAAGTAGCGAGGAAGCCGCTACAAAC**GGC**ACCGTCTAGAATCCT A>G	
TS3#14	A1: TCACCAAGAAAAGTAGCGAAGAAGCCGCTACAAAC**GGC**ACCGTCTAGAATCCT A>G	Bia
	A2: TCACCAAGAAAAGTAGCGAGGAAGCCGCTACAAAC**GGC**ACCGTCTAGAATCCT A>G	
TS3#16	A1: TCACCAAGAAAAGTAGCGAAGAAGCCGCTACAAAC**GGC**ACCGTCTAGAATCCT A>G	Bia
	A2: TCACCAAGAAAAGTAGCGAGGAAGCCGCTACAAAC**GGC**ACCGTCTAGAATCCT A>G	
TS3#18	A1: TCACCAAGAAAAGTAGCGAAAAAGCCGCTACAAAC**GGC**ACCGTCTAGAATCCT WT	Het
	A2: TCACCAAGAAAAGTAGCGAAGAAGCCGCTACAAAC**GGC**ACCGTCTAGAATCCT A>G	
TS3#19	A1: TCACCAAGAAAAGTAGCGAGGAAGCCGCTACAAAC**GGC**ACCGTCTAGAATCCT A>G	Bia
	A2: TCACCAAGAAAAGTAGCGAAGAAGCCGCTACAAAC**GGC**ACCGTCTAGAATCCT A>G	
TS3#21	A1: TCACCAAGAAAAGTAGCGAGGGAGCCGCTACAAAC**GGC**ACCGTCTAGAATCCT A>G	Bia
	A2: TCACCAAGAAAAGTAGCGGGGGAGCCGCTACAAAC**GGC**ACCGTCTAGAATCCT A>G	
TS3#23	A1: TCACCAAGAAAAGTAGCGAAAAAGCCGCTACAAAC**GGC**ACCGTCTAGAATCCT WT	Het
	A2: TCACCAAGAAAAGTAGCGAGGAAGCCGCTACAAAC**GGC**ACCGTCTAGAATCCT A>G	

ABE8e-hAAG-TS4

ABE8e-hAAG	Ref:ACGCATGGATAAACAAGAAGAAGACGATGAGCATT**AGG**GTTCCTTAACCAGTT	Allelic variation
TS4#01	A1: ACGCATGGATAAACAAGAAGGAGACGATGAGCATT**AGG**GTTCCTTAACCAGTT A>G	Hom
	A2: ACGCATGGATAAACAAGAAGGAGACGATGAGCATT**AGG**GTTCCTTAACCAGTT A>G	
TS4#04	A1: ACGCATGGATAAACAAGAAGAAGACGATGAGCATT**AGG**GTTCCTTAACCAGTT WT	Het
	A2: ACGCATGGATAAACAAGAGGGAGACGATGAGCATT**AGG**GTTCCTTAACCAGTT A>G	
TS4#05	A1: ACGCATGGATAAACAAGAAGGGGACGATGAGCATT**AGG**GTTCCTTAACCAGTT A>G	Bia
	A2: ACGCATGGATAAACAAGAAGGAGACGATGAGCATT**AGG**GTTCCTTAACCAGTT A>G	
TS4#07	A1: ACGCATGGATAAACAAGAAGAAGACGATGAGCATT**AGG**GTTCCTTAACCAGTT WT	Het
	A2: ACGCATGGATAAACAAGAAGGAGACGATGAGCATT**AGG**GTTCCTTAACCAGTT A>G	
TS4#08	A1: ACGCATGGATAAACAAGAGGAAGACGATGAGCATT**AGG**GTTCCTTAACCAGTT A>G	Bia
	A2: ACGCATGGATAAACAAGAAGGAGACGATGAGCATT**AGG**GTTCCTTAACCAGTT A>G	
TS4#10	A1: ACGCATGGATAAACAAGAAGGAGACGATGAGCATT**AGG**GTTCCTTAACCAGTT A>G	Bia
	A2: ACGCATGGATAAACAAGAGGAAGACGATGAGCATT**AGG**GTTCCTTAACCAGTT A>G	
TS4#11	A1: ACGCATGGATAAACAAGAAGGAGACGATGAGCATT**AGG**GTTCCTTAACCAGTT A>G	Bia
	A2: ACGCATGGATAAACAAGAGGGGGACGATGAGCATT**AGG**GTTCCTTAACCAGTT A>G	
TS4#12	A1: ACGCATGGATAAACAAGAAGAAGACGATGAGCATT**AGG**GTTCCTTAACCAGTT WT	Het
	A2: ACGCATGGATAAACAAGAAGGAGACGATGAGCATT**AGG**GTTCCTTAACCAGTT A>G	
TS4#13	A1: ACGCATGGATAAACAAGAGGGAGACGATGAGCATT**AGG**GTTCCTTAACCAGTT A>G	Bia

	A2: ACGCATGGATAAACAAGAGGGGGACGATGAGCATTAGGGTTCCTTAACCAGTT A>G	
TS4#14	A1: ACGCATGGATAAACAAGGGGGAGACGATGAGCATTAGGGTTCCTTAACCAGTT A>G<br>A2: ACGCATGGATAAACAAGGGGGAGACGATGAGCATTAGGGTTCCTTAACCAGTT A>G	Hom
TS4#15	A1: ACGCATGGATAAACAAGAAGGGGACGATGAGCATTAGGGTTCCTTAACCAGTT A>G<br>A2: ACGCATGGATAAACAAGAAGGAGACGATGAGCATTAGGGTTCCTTAACCAGTT A>G	Bia
TS4#16	A1: ACGCATGGATAAACAAGAGGGGGACGATGAGCATTAGGGTTCCTTAACCAGTT A>G<br>A2: ACGCATGGATAAACAAGAAGGAGACGATGAGCATTAGGGTTCCTTAACCAGTT A>G	Bia
TS4#17	A1: ACGCATGGATAAACAAGAGGGAGACGATGAGCATTAGGGTTCCTTAACCAGTT A>G<br>A2: ACGCATGGATAAACAAGAAGGAGACGATGAGCATTAGGGTTCCTTAACCAGTT A>G	Bia
TS4#19	A1: ACGCATGGATAAACAAGAGGGGGACGATGAGCATTAGGGTTCCTTAACCAGTT A>G<br>A2: ACGCATGGATAAACAAGAAGGAGACGATGAGCATTAGGGTTCCTTAACCAGTT A>G	Bia
TS4#20	A1: ACGCATGGATAAACAAGAAGGAGACGATGAGCATTAGGGTTCCTTAACCAGTT A>G<br>A2: ACGCATGGATAAACAAGAGGAAGACGATGAGCATTAGGGTTCCTTAACCAGTT A>G	Bia
TS4#21	A1: ACGCATGGATAAACAAGAGGGGGACGATGAGCATTAGGGTTCCTTAACCAGTT A>G<br>A2: ACGCATGGATAAACAAGAAGGAGACGATGAGCATTAGGGTTCCTTAACCAGTT A>G	Bia
TS4#22	A1: ACGCATGGATAAACAAGAGGGAGACGATGAGCATTAGGGTTCCTTAACCAGTT A>G<br>A2: ACGCATGGATAAACAAGGGGGGGACGATGAGCATTAGGGTTCCTTAACCAGTT A>G	Bia
TS4#23	A1: ACGCATGGATAAACAAGGGGGAGACGATGAGCATTAGGGTTCCTTAACCAGTT A>G<br>A2: ACGCATGGATAAACAAGAGGGGGACGATGAGCATTAGGGTTCCTTAACCAGTT A>G	Bia
TS4#02	A1: ACGCATGGATAA--------AAGACGATGAGCATTAGGGTTCCTTAACCAGTT -8bp<br>A2: ACGCATGGATAAACAAGAGGGAGACGATGAGCATTAGGGTTCCTTAACCAGTT A>G	Bia

ABE8e-hAAG-TS5

ABE8e-hAAG	Ref:TAGATCCATCACATCTACTAAGACACGCGCCGCCATGATCGAGGTAATTAAGG	Allelic variation
TS5#01	A1: TAGATCCATCACATCTACTGAGACACGCGCCGCCATGATCGAGGTAATTAAGG A>G<br>A2: TAGATCCATCACATCTACTGAGACACGCGCCGCCATGATCGAGGTAATTAAGG A>G	Hom
TS5#05	A1: TAGATCCATCACATCTACTAAGACACGCGCCGCCATGATCGAGGTAATTAAGG WT<br>A2: TAGATCCATCACATCTACTGAGACACGCGCCGCCATGATCGAGGTAATTAAGG A>G	Het
TS5#07	A1: TAGATCCATCACATCTACTAAGACACGCGCCGCCATGATCGAGGTAATTAAGG WT<br>A2: TAGATCCATCACATCTACTGGGACACGCGCCGCCATGATCGAGGTAATTAAGG A>G	Het
TS5#08	A1: TAGATCCATCACATCTACTGGGACACGCGCCGCCATGATCGAGGTAATTAAGG A>G<br>A2: TAGATCCATCACATCTACTGGGACACGCGCCGCCATGATCGAGGTAATTAAGG A>G	Hom
TS5#10	A1: TAGATCCATCACATCTACTGAGACACGCGCCGCCATGATCGAGGTAATTAAGG A>G<br>A2: TAGATCCATCACATCTACTGGGGCACGCGCCGCCATGATCGAGGTAATTAAGG A>G	Bia
TS5#11	A1: TAGATCCATCACATCTACTAAGACACGCGCCGCCATGATCGAGGTAATTAAGG WT<br>A2: TAGATCCATCACATCTACTGGGACACGCGCCGCCATGATCGAGGTAATTAAGG A>G	Het
TS5#13	A1: TAGATCCATCACATCTACTGGGGCACGCGCCGCCATGATCGAGGTAATTAAGG A>G<br>A2: TAGATCCATCACATCTACTGAGACACGCGCCGCCATGATCGAGGTAATTAAGG A>G	Bia
TS5#14	A1: TAGATCCATCACATCTACTGAGACACGCGCCGCCATGATCGAGGTAATTAAGG A>G<br>A2: TAGATCCATCACATCTACTGGGACACGCGCCGCCATGATCGAGGTAATTAAGG A>G	Bia
TS5#15	A1: TAGATCCATCACATCTACTGGGACACGCGCCGCCATGATCGAGGTAATTAAGG A>G<br>A2: TAGATCCATCACATCTACTGAGACACGCGCCGCCATGATCGAGGTAATTAAGG A>G	Bia
TS5#16	A1: TAGATCCATCACATCTACTGAGACACGCGCCGCCATGATCGAGGTAATTAAGG A>G	Bia

	A2: TAGATCCATCACATCTACTGAGGCACGCGCCGCCA**TGA**TCGAGGTAATTAAGG A>G	
TS5#17	A1: TAGATCCATCACATCTACTAAGACACGCGCCGCCA**TGA**TCGAGGTAATTAAGG WT<br>A2: TAGATCCATCACATCTACTGAGACACGCGCCGCCA**TGA**TCGAGGTAATTAAGG A>G	Het
TS5#18	A1: TAGATCCATCACATCTACTGAGACACGCGCCGCCA**TGA**TCGAGGTAATTAAGG A>G<br>A2: TAGATCCATCACATCTACTAGGGCACGCGCCGCCA**TGA**TCGAGGTAATTAAGG A>G	Bia
TS5#19	A1: TAGATCCATCACATCTACTGAGACACGCGCCGCCA**TGA**TCGAGGTAATTAAGG A>G<br>A2: TAGATCCATCACATCTACTGAGACACGCGCCGCCA**TGA**TCGAGGTAATTAAGG A>G	Hom
TS5#20	A1: TAGATCCATCACATCTACTGAGACACGCGCCGCCA**TGA**TCGAGGTAATTAAGG A>G<br>A2: TAGATCCATCACATCTACTGGGACACGCGCCGCCA**TGA**TCGAGGTAATTAAGG A>G	Bia
TS5#21	A1: TAGATCCATCACATCTACTAAGACACGCGCCGCCA**TGA**TCGAGGTAATTAAGG WT<br>A2: TAGATCCATCACATCTACTAGGACACGCGCCGCCA**TGA**TCGAGGTAATTAAGG A>G	Het
TS5#22	A1: TAGATCCATCACATCTACTAGGACACGCGCCGCCA**TGA**TCGAGGTAATTAAGG A>G<br>A2: TAGATCCATCACATCTACTAAGGCACGCGCCGCCA**TGA**TCGAGGTAATTAAGG A>G	Bia
TS5#23	A1: TAGATCCATCACATCTACTGAGACACGCGCCGCCA**TGA**TCGAGGTAATTAAGG A>G<br>A2: TAGATCCATCACATCTACTGAGGCACGCGCCGCCA**TGA**TCGAGGTAATTAAGG A>G	Bia

Sequence S1. The sequence of rUNG and hUNG.

>rUNG

ATGGCTCCACCACTTCCACCAACAGCTCCAAAGACGATCGCCGACTACCTCATCAGGCCGTCCAAGAGGCT
TAGGCCAACATCTCCAGCTCCAGCCGCTGCTGCCTCTGCTCCACTCTCTTCTAGTTCGCTCTCCCCGGAACA
AAGGCGCAGGGCCGATACAAATCTCGCCCTCGCCAGAGCTAGACGCCATCTCAGACTTGCCGAGTCTAAG
GCTTCTGGCGGCACAGCCAAGCTCGAGGAACTCCTCGTTGAGGAAACCTGGCTCGAAGCGTTGCCAGGC
GAGCTGCATAAGCCATACGCTCTCGAGCTTTGCCGCTTCGTGGCCCATGAGAGGCTTCATTCTCCGGTGCC
AGTGTACCCACCACCGCACCTCGTTTTCCATGCGCTCCATGCCACACCGTTCGATCGCGTGAAGGCCGTGA
TCATCGGACAGGACCCATATCATGGCCCAGGCCAAGCTATGGGCCTCAGCTTTTCTGTGCCAGAGGGCAT
CAAGATCCCGTCCAGCCTCGCGAACATCTTCAAAGAGCTTCAGAAGGACCTCGGCTGCACCGTGCCATCTC
ATGGCAATCTTGAGAGGTGGGCCGTGCAGGGCGTGCTCATGCTCAATACTGTGCTCACCGTGCGCGAGC
ACCAGGCCAATAGCCATGCTAAGAAAGGCTGGGAGCAGTTCACCGACGCCGTCATCAAGACCATCAGCCT
CAAGAAGTCCGGCATCGTTTTCATCCTCTGGGGCAACAGCGCCCAGGCTAAGACCAGGCTCATCGACGAG
ACAAAGCACCACATCCTCAAGAGCGCCCATCCATCTGGCCTTTCTGCTTCCAGAGGCTTCTTCGGCTGCCG
CCACTTCTCCAAGACCAACCAGATTCTCGAGCGCCTCGGCCTCTCTGCCATTGATTGGCAGCTC

>hUNG

ATGTTTGGCGAGTCCTGGAAGAAGCACCTCTCCGGCGAATTCGGCAAGCCGTACTTCATCAAGCTCATGGG
CTTCGTGGCCGAGGAACGCAAGCACTACACAGTGTACCCACCGCCGCACCAGGTGTTCACCTGGACACAGA
TGTGCGACATCAAGGACGTGAAGGTGGTGATCCTCGGACAGGACCCATACCACGGACCAAATCAAGCCCAC
GGCCTCTGCTTCTCTGTGCAGAGGCCAGTTCCACCACCACCGAGCCTCGAGAACATCTACAAAGAGCTGTC
CACCGACATCGAGGACTTCGTGCATCCAGGCCATGGCGATCTTTCTGGCTGGGCCAAACAAGGCGTGCTCC
TCCTCAATGCCGTGCTCACAGTTAGGGCCCATCAGGCCAACAGCCACAAAGAAAGAGGCTGGGAGCAGTT
CACCGACGCCGTTGTGTCTTGGCTCAACCAGAACTCCAACGGCCTCGTGTTCTTGCTCTGGGGCTCTTACGC
CCAGAAGAAGGGCTCTGCCATCGACCGCAAGAGGCACCATGTGCTCCAAACAGCTCATCCGTCACCGCTCT
CAGTGTACAGGGGCTTCTTCGGCTGTCGCCACTTCTCCAAGACCAACGAGCTGCTCCAGAAGTCCGGCAAG
AAGCCGATCGACTGGAAAGAGCTT

Sequence S2. The sequence of hAAG and EndoV.

>hAAG

ATGAAGGGCCATCTCACAAGGCTCGGCCTCGAGTTCTTCGATCAACCAGCTGTTCCTCTCGCCAGGGCCTTCCTCGGCC
AAGTGCTCGTTAGAAGGCTCCCGAATGGCACCGAACTCCGCGGCAGAATCGTTGAGACAGAGGCCTATCTCGGCCCAGA
GGATGAGGCCGCTCATTCTAGAGGCGGCAGACAGACACCTCGCAACCGCGGCATGTTTATGAAGCCGGGCACGCTCTAC
GTGTACATTATCTACGGCATGTATTTCTGCATGAACATCTCCAGCCAAGGCGACGGCGCTTGCGTTCTCCTTAGAGCCC
TTGAGCCACTCGAGGGCCTCGAAACCATGAGGCATGTGAGGTCCCAGCTGAGGAAGGGCACAGCTTCTAGGGTGCTCAA
GGATCGCGAGTTGTGCAGCGGACCGTCCAAGCTCTGTCAAGCCCTCGCCATCAACAAGTCCTTCGACCAGAGAGATCTC
GCCCAGGACGAGGCTGTTTGGCTTGAAAGAGGCCCACTCGAGCCATCTGAGCCAGCCGTTGTTGCTGCTGCTAGAGTTG
GCGTTGGCCATGCTGGCGAATGGGCTAGAAAGCCGCTCCGCTTCTATGTGAGAGGCTCCCCATGGGTGTCAGTGGTGGA
TAGAGTGGCCGAGCAAGA

> EndoV

ATGGATCTCGCTTCTCTCAGGGCCCAGCAGATCGAGCTTGCCTCTTCTGTGATCCGCGAGGACAGGCTCGATAAGGACC
CGCCGGATCTTATTGCCGGCGCTGATGTTGGCTTTGAGCAAGGCGGCGAGGTTACAAGAGCCGCCATGGTGCTCCTCAA
GTACCCATCTCTCGAGCTGGTCGAGTACAAGGTGGCCAGGATCGCCACCACCATGCCGTATATTCCAGGCTTCCTCAGC
TTCCGCGAGTACCCTGCACTTCTCGCTGCTTGGGAGATGCTCAGCCAAAAGCCGGACCTCGTGTTCGTGGATGGCCACG
GCATTTCTCATCCAAGAAGGCTCGGCGTCGCCAGCCATTTCGGCCTTCTCGTGGATGTTCCAACCATCGGCGTGGCCAA
GAAGAGGCTCTGCGGCAAGTTTGAGCCACTCTCTTCAGAGCCAGGCGCTCTCGCTCCGCTTATGGATAAGGGCGAGCAA
CTCGCCTGGGTGTGGCGTTCTAAGGCCAGGTGCAACCCGCTCTTTATCGCCACAGGCCATAGGGTGTCAGTGGATTCTG
CCCTCGCTTGGGTGCAGAGATGCATGAAGGGCTACAGACTCCCAGAGCCAACCAGATGGGCCGATGCTGTTGCTTCTGA
GAGGCCAGCCTTCGTGAGGTACACCGCCAATCAGCCA
